# Asymmetric Evolution of Antennal Cell Types Underlies a Derived Ammonia‐Sensing Logic for Ecological Adaptation in *Bactrocera dorsalis*

**DOI:** 10.1002/advs.77168

**Published:** 2026-08-17

**Authors:** Wei Liu, Shuai Zhang, Jie Zhang, Zhen Tian, Yuanbo Xue, Ling Yang, Jun Xu, Guirong Wang

**Affiliations:** ^1^ Shenzhen Branch, Guangdong Laboratory of Lingnan Modern Agriculture, Key Laboratory of Synthetic Biology, Ministry of Agriculture and Rural Affairs, Agricultural Genomics Institute at Shenzhen Chinese Academy of Agricultural Sciences Shenzhen China; ^2^ State Key Laboratory of Plant Trait Design, Center For Excellence in Molecular Plant Sciences Chinese Academy of Sciences Shanghai China; ^3^ University of Chinese Academy of Sciences Beijing China; ^4^ Hubei Key Laboratory of Biological Resources Protection and Utilization, College of Forestry and Horticulture HuBei Min Zu University Enshi China; ^5^ School of Forestry, Key Laboratory of Sustainable Forest Ecosystem Management‐Ministry of Education Northeast Forestry University Harbin China; ^6^ Shanxi Key Laboratory of Efficient Cultivation of Forest Resources, College of Forestry Shanxi Agricultural University Jinzhong China

**Keywords:** *amt*, antenna, *Bactrocera dorsalis*, ecological adaptation, snRNA‐seq

## Abstract

Olfaction is crucial for insects adapting to agroecosystems and becoming significant crop pests, but how the antenna, a multicellular olfactory organ, is systematically organized at the cellular and molecular levels is not well understood. This study focuses on tephritids, particularly *Bactrocera dorsalis*, due to its global invasiveness and severe agricultural impact. We conducted comparative single‐nucleus transcriptomics of the antennal olfactory organ in *B. dorsalis* and *Drosophila melanogaster*, revealing uneven transcriptomic divergence across cell types: structural cells showed higher cross‐species similarity, while sensory neuronal populations and receptor expression were more divergent. Notably, in *B. dorsalis*, ammonia detection involves an additional candidate neuronal population with *BdorAmt/BdorOrco* transcript co‐detection, contrasting with *D. melanogaster*, where ammonia detection is mediated by *Amt*‐expressing neurons independently of *Orco*. This derived molecular sensing logic helps meet the ecological demands of *B. dorsalis*, in which females use ammonia as an important cue to locate bird droppings as a potential supplementary nutritional resource. Our results suggest that cell‐type‐dependent transcriptomic divergence may represent one important route through which insect olfactory systems adapt, with neuronal diversification contributing to molecular sensing pathways tailored to specific ecological demands.

## Graphical abstract

Comparative antennal single‐nucleus transcriptomics reveals cell‐type‐dependent transcriptomic divergence in *Bactrocera dorsalis*, with similar structural cells but divergent sensory neurons. This neuronal diversification is associated with a distinct ammonia‐sensing pathway, linking female attraction to bird‐dropping‐associated cues with nutritional and reproductive ecological demands.

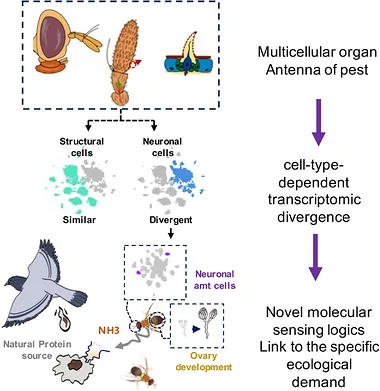

## 1 Introduction

Agroecosystems are specialized, human‐managed ecosystems that serve as a critical source of food [1]. As a typical novel ecosystem, crop phenotypes have diverged from those in natural environments due to long‐term cultivation. Meanwhile, through crop cultivation, human activities such as tillage, irrigation, and fertilization have profoundly altered original ecological processes [2]. In response to the features of this novel ecosystem, insects have shown remarkable adaptability through prolonged interactions with domesticated crops and during their worldwide expansion, eventually evolving into economically significant pests [3]. To occupy new ecological niches within novel ecosystems, insects have evolved adaptive traits across multiple dimensions, including behavior, physiology, and morphology. For example, fruit‐associated Dipterans occupy distinct resource niches according to fruit condition and host range. *Drosophila melanogaster* uses fermentation volatiles produced by yeasts and other microbes to locate decaying or fermenting fruits [4]. *D. suzukii* has adapted to intact ripening fruits through altered sensory preferences together with a serrated ovipositor [5]. *Bactrocera dorsalis*, in turn, uses host‐specific volatile compounds emitted by different fruits to locate and exploit a broad range of hosts [6]. Beyond host use, niche adaptation may also extend to the strategies by which insects meet the protein demands of reproduction. Yeasts associated with fermenting fruits provide a major source of dietary protein for *D. melanogaster*, and mated females increase their preference for yeast to meet reproductive nutritional demands [7]. Notably, early field observations documented adult tephritid fruit flies feeding on nitrogen‐rich bird droppings, suggesting an additional strategy for nutritional supplementation [6, 8, 9, 10]. Together, these examples illustrate how behavioral adaptations, often supported by sensory and morphological changes, enable fruit‐associated insects to exploit diverse host and nutritional resources.

The adaptation of olfactory behaviors is central to insects’ ability to occupy new ecological niches, largely because numerous olfaction‐related ecological factors are widespread in agroecosystems. For example, insects rely on pheromones released by conspecifics to locate mates and reproduction sites [11]. Other insects or microbes may release defensive or alarm signals that affect insect survival and decision‐making [12]. Kairomones emitted by crops provide cues for feeding and oviposition [13]. Additionally, human activities, such as deploying synthetic pheromones or applying chemical insecticides, can strongly influence insect behavior through attraction or repellence [14, 15]. These olfaction‐related ecological factors generally act on the insects’ olfactory organs (e.g., the antennae), which transduce chemical signals into electrical signals to the brain and thereby induce corresponding behaviors [16]. Therefore, current research has generally followed the “chemicals‐olfactory receptors‐ behavior” framework, with olfactory receptors and associated sensory neurons as the primary focus. However, the antenna is not a simple “receptor–neuron” assembly. It is a relatively complex, multicellular organ composed of epithelium, supporting cells, glia, and neurons [17, 18, 19, 20, 21]. Consequently, the reception and amplification of olfactory signals are not limited to receptor activation alone but are modulated by molecular networks distributed across multiple cell types [22, 23, 24].

Single‐cell RNA sequencing (scRNA‐seq), a technology for cell typing based on molecular profiles widely used in mammalian neurobiology [25, 26, 27, 28, 29], has recently been applied to *Drosophila*. This approach is particularly helpful for distinguishing complex neuronal populations, such as olfactory projection neurons located in the brain [30]. The technique was subsequently adapted to single‐nucleus RNA sequencing (snRNA‐seq) to overcome challenges in cell isolation caused by the insect's rigid chitinous exoskeleton [18]. Building on these advances, the Fly Cell Atlas (FCA) was systematically established, covering 17 tissues and identifying more than 250 cell types [19]. Both scRNA‐seq and snRNA‐seq offer substantially increased resolution, enabling the identification of key transcription factors in specific olfactory neurons [17]. Furthermore, at single‐cell resolution, these techniques facilitate the reconstruction of transcriptional programs and temporally ordered regulatory networks underlying antennal development [18]. This progress will yield highly informative data and enhance our understanding of olfactory circuits and synaptogenesis. Beyond model insects, scRNA‐seq and snRNA‐seq are increasingly being extended to diverse non‐model systems to address questions spanning development, physiology, behavior, and ecological adaptation. For example, in social insects, single‐cell approaches have revealed cellular programs associated with social reprogramming and division of labor, providing insights into the cellular organization of insect superorganisms [31, 32].

As scRNA‐seq and snRNA‐seq have been successfully applied to *Drosophila* and non‐model insects, it gives us the opportunity to understand how olfactory‐related ecological factors affect the antennae of pests in agroecosystems from a cellular perspective. This methodology would be useful for further elucidating the molecular and cellular basis of the behavior modification of the pest. The tephritid fruit flies have long been recognized as major pests in agroecosystems worldwide, and their life history is highly reliant on their detection of semiochemicals in the environment [33, 34]. Furthermore, these olfactory cues, which have been developed into technologies for insect behavior modification, have long been proven to be effective control strategies in practice [35, 36, 37]. Therefore, we establish an snRNA‐seq atlas of the antennae of *B. dorsalis* in this study, a representative pest species among tephritids that cause damage globally. Further analysis utilizing the information of snRNA‐seq of *D. melanogaster* to support cell typing and detailed functional annotation for *B. dorsalis*. Cross‐species comparisons of antennal cells between *B. dorsalis* and *D. melanogaster* are used to uncover key information related to their olfactory ecological adaptations. Finally, the further functional validations focus on specific neuronal populations correlated with behavioral divergence across these two species. In *B. dorsalis*, ammonia‐mediated attraction is female‐specific and involves both a conserved *Amt/Rh50* co‐expressing neuron, similar to *D. melanogaster*, and an additional *Amt/Orco* expressing neuron that is unique to *B. dorsalis*; notably, both *Amt* and *Orco* are required for female attraction to ammonia, contrasting with *D. melanogaster* where *Orco* does not play a role. These findings highlight the divergence of neuronal types and functions at the cellular level and their significance in adapting to dynamic ecological environments.

## 2 Results

### 2.1 A Single‐Nucleus Atlas of the Adult Antenna of *B. dorsalis*

The insect antenna is typically composed of sensory neurons, epithelial cells, supporting cells, muscle cells, and hemocyte. Using nuclear isolation followed by fluorescence‐activated cell sorting (FACS), we obtained high‐quality single‐nucleus suspensions from *B. dorsalis*. Library preparation and sequencing were performed using the 10× Genomics platform (Figure 1A) (Data S9). We successfully recovered 16 939 nuclei from 60 male and 60 female antennae, identified 30 distinct clusters representing 11 support cells (including 4 glia cells), 11 neurons cells, 2 epithelial cells, muscle cell, hemocyte, and 4 unannotated cells (Figure 1B). To assess the robustness of the clustering results, we additionally performed clustering using the Leiden algorithm with the same Harmony embeddings and clustering resolution. The resulting clusters were highly consistent with those obtained by the Louvain algorithm (ARI = 0.88), indicating that the identified cell populations were robust to the choice of community detection algorithm (Figure S1). The antenna neuron cell clusters are defined by the expression of *Orco*, and the olfactory receptor, johnston organ neuron cluster express *Dhc1* and *Dhc93AB* [38], lamina monopolar neuron L4 express *nAChRalpha6*, *Dop1R1*, and *5‐HT1A* [39, 40, 41]. The epithelial cells express *NAAT1‐1* and *grh*. The 11 support cell clusters express the markers of *ance‐3*, *Obp69a*, *Su(H)*, *sv‐1*, *Obp19a‐2*, *dve*, *Desat1*, *Amt*, *GluClalpha*, *Obp84a‐2*, *lush*, *Obp83a‐2*, *Obp59a*, *Mdr65*, *repo*, *Drgx*, *apt‐1*, *svp‐1*, *nub*, *sens‐2* (Figure 1C and Data S1 *)*.

**FIGURE 1:**
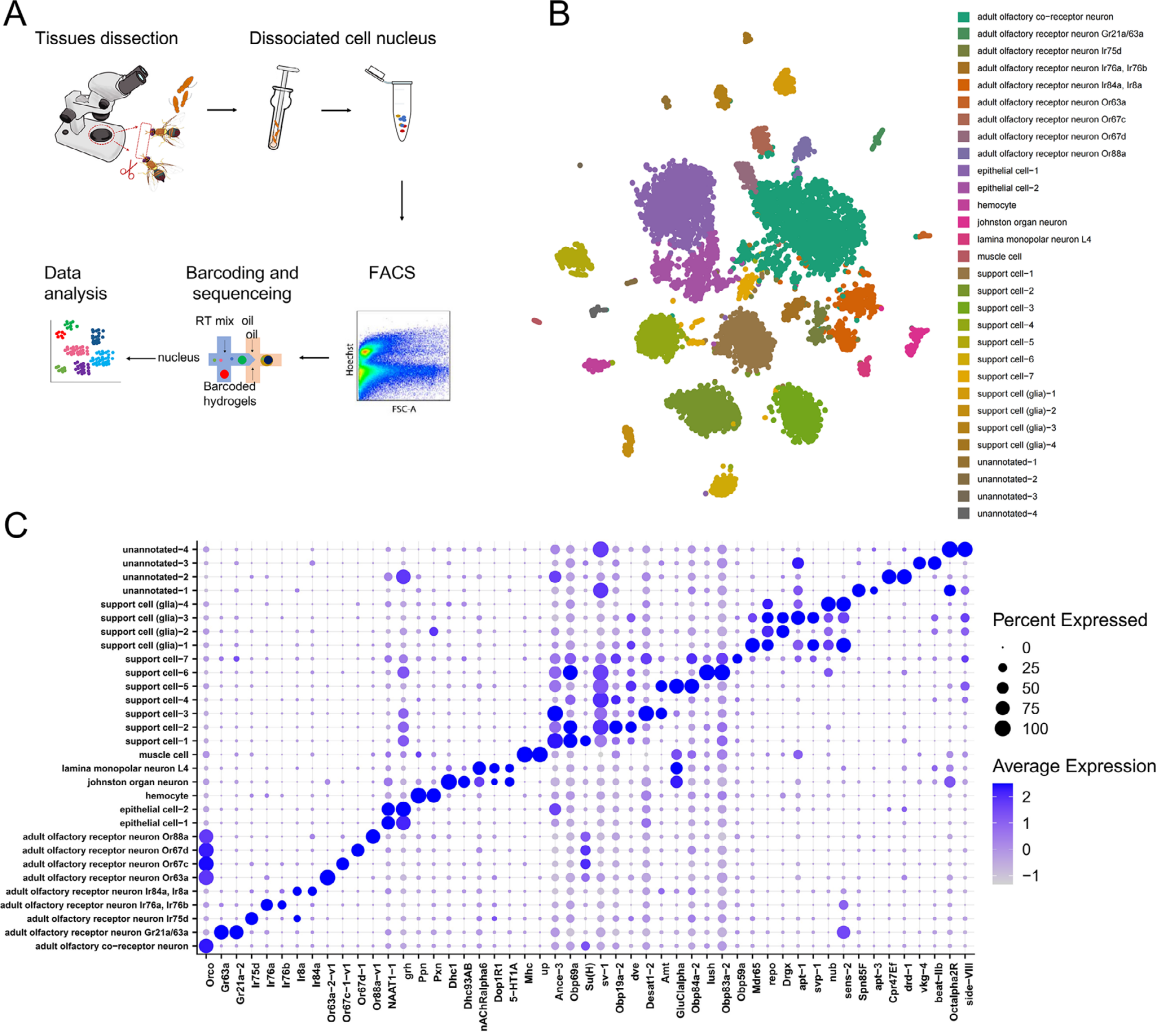
Construction of a single‐nucleus atlas for the antenna of *B. dorsalis*. (A) Workflow of snRNA‐seq analysis of the *B. dorsalis* adult antenna. (B) A single t‐SNE map of the antenna contains 30 distinct cell clusters that were annotated on the t‐SNE map. (C) Expression levels and percentage of cells expressing the marker genes in each cluster are shown as a dot plot.

To investigate the expression and distribution of sensory receptors in the antennae, we analyzed the expression patterns of olfactory receptors, gustatory receptors, and ionotropic receptors in antennal single‐nucleus data using previously obtained genome sequencing and annotation information [42]. The results demonstrated that a total of 62 olfactory receptors, 14 gustatory receptors and 22 ionotropic receptors showed specific expression patterns in the antennal single‐nucleus atlas (Data S2). Among these receptors, *Or63s*, *Or67s*, *Or88s* was characterized by high expression levels and a large number of expressing cells, indicating potential crucial roles of olfactory receptors in the antenna for the ecological adaptation of *B. dorsalis*.

We next systematically examined sexually dimorphic expression patterns of antennal olfactory receptors. The analysis identified *Or13a‐2*, *Or2‐1‐v1*, *Or43b*, *Or49b*, *Or59a‐2‐v1*, *Or63a‐2‐v1*, *Or67d‐5*, *Or69a‐1*, *Or74a*, *Or7a‐13‐v2* and *Or92a* as female‐biased, and *Or59a‐2‐v2*, *Or43a‐1*, *Or7a‐1‐v1*, *Or7a‐13‐v1*, as male‐biased. *Or67cs*, however, was highly expressed in both females and males and exhibited no sexually dimorphic expression. *Or82a* is exclusively expressed in females within the adult olfactory co‐receptor neuron cluster, but is male‐specific in the johnston organ neuron cluster (Figure S2). This demonstrates the divergent sex‐biased expression patterns of the same receptor across different cell populations. The sex‐biased expression patterns reported here represent differences between unmated 12‐day‐old adults; potential mating‐induced transcriptional changes were not assessed.

To investigate the transcription factors (TFs) that may contribute to antenna differentiation and function, we applied SCENIC (single‐cell regulatory network inference and clustering) to reveal TF‐centered gene co‐expression networks [43] for the simultaneous reconstruction of gene‐regulatory networks and identification of cell states. Using a gene‐correlation network followed by motif‐based filtration, SCENIC refines the modules (regulons) to retain only the potential direct regulatory targets of each transcription factor. A total of 65 TFs were identified through this approach, (Figure S3). Among the TFs, *EcR*, *Rbp6*, *bru3*, *onecut*, *Kdm4B*, *acj6*, *Mef2*, *dl*, *sr*, *Sidpn*, *Rfx*, and *Xbp1* are highly and specifically expressed in 11 antenna neuron cell clusters. *Mid*, *NFAT*, *Pdp1*, *vri*, *net*, *dysf*, *CG10348*, *gem*, *kay*, *Rel*, *CHES‐1like*, *Mitf*, *foxo*, *CRebA*, *Hnf4* and *grh* are expressed in non‐neuron cell clusters. *Repo* is specifically expressed in 4 support cell (glia) cell clusters. The SCENIC could infer multiple downstream target genes. For example, among the *acj6* target genes are *ab* and *amon*, the *Unc‐4* target genes are *Znt35C* and *MFS9*, the *Hnf4* target genes are *dsb* and *mdy* (Figure S3 and Data S3 *). In D. melanogaster*, *Acj6* and *Unc‐4* are required for olfactory neuronal development and function, whereas *Hnf4* regulates cellular lipid homeostasis. Their inferred regulons in *B. dorsalis* antennal cells suggest potential roles in neuronal differentiation and metabolic regulation, respectively. Altogether, our analysis provides a list of candidate TFs that may control antenna cells maintenance, morphology, and physiological function, which does offer an entrance into future research.

### 2.2 Cross‐species Comparative Analysis of Antennal Cell Cluster Functions in *B. dorsalis*

For a detailed functional profiling of antennal cells in *B. dorsalis*, we conducted a cross‐species analysis of antennal cells between *B. dorsalis* and *D. melanogaster*. Based on cell cluster marker genes identified from single‐nucleus sequencing of *Drosophila* and mosquito antennae, we classified the cell clusters in *B. dorsalis* into four major categories: neurons (marker genes: *brp*, *para*, *Syt1*), epithelial cells (marker gene: *NAAT1‐1*, *Grh*), support cells (marker genes: *sv‐1*, *Ance‐3*), and glial cells (marker genes: *repo*, *sens‐2*) (Figure 2A). These four major categories appear well‐separated in the t‐SNE map projection, indicating their highly distinct cellular identities (Figure 2B). We used the Self‐Assembling Manifold mapping (SAMap) algorithm [44] to map our *B. dorsalis* single‐nucleus transcriptomes with snRNA‐seq data from *D. melanogaster* [19]. Using this method, SAMap generated a combined manifold exhibiting high cross‐species alignment (Figure S4A). We then quantified the mapping strength between cell types by calculating an alignment score, defined as the average number of mutual nearest cross‐species neighbors per cell relative to the maximum possible. This score is represented by edge width in Figure 2C. Based on this metric, we generated a Sankey plot illustrating the correspondence between *B. dorsalis* and *D. melanogaster* antennal cell clusters. Neuronal, epithelial, supportive, glial, muscular, and hemocytic cells exhibit a collinear relationship with the major cell populations in *D. melanogaster* (Figure 2C).

**FIGURE 2:**
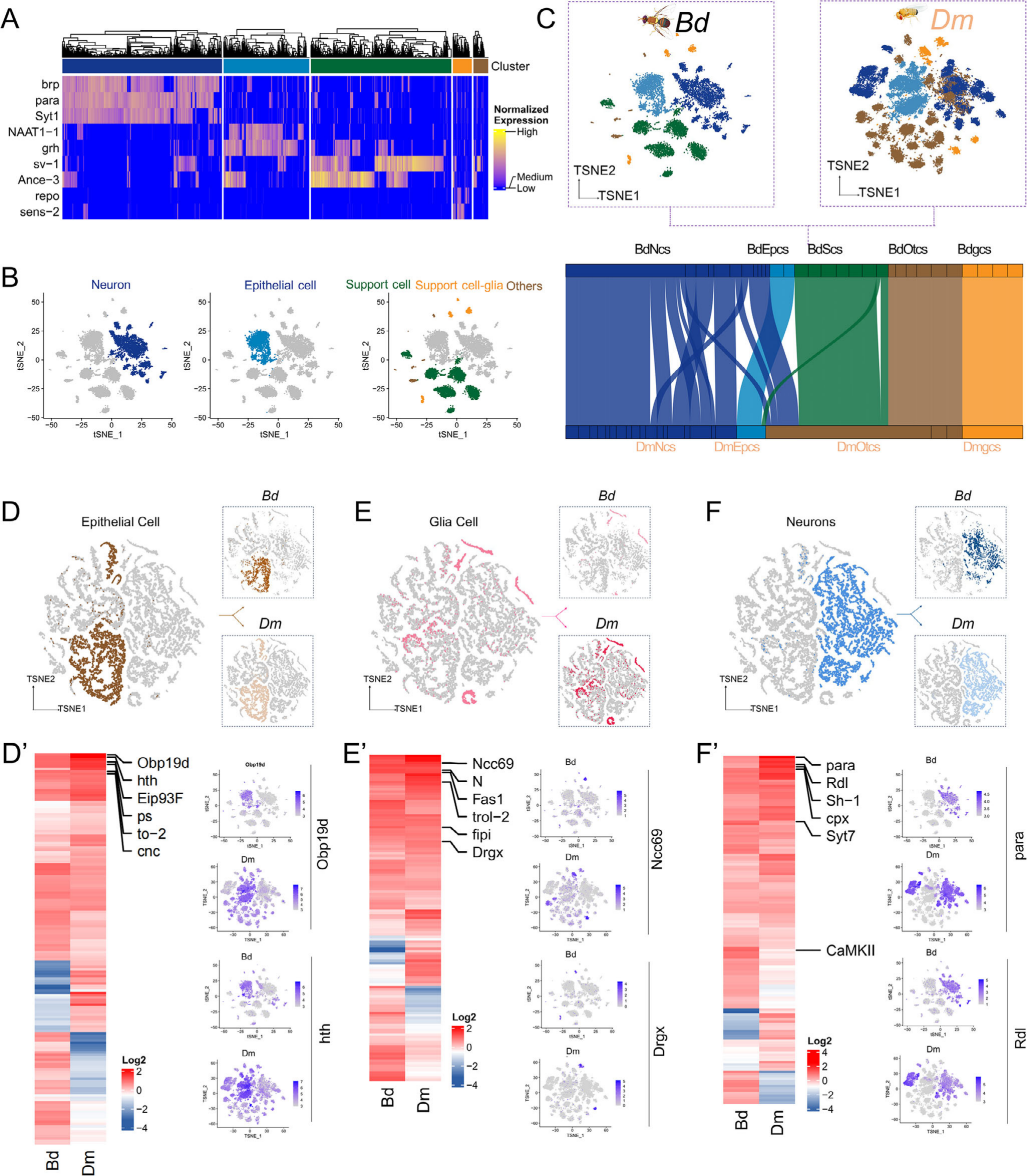
Cross‐species analysis of *B. dorsalis* and *D. melanogaster* antenna using SAMap. (A) Heatmap of antenna cells within clusters that express cell‐type markers according to normalized expression. (B) Distribution of the major cell clusters on the t‐SNE map. (C) Sankey plot summarizing the cell‐type mappings. Edges with alignment scores < 0.2 were omitted. (D–F’) Comparative analysis of three functional cell clusters between *B. dorsalis* and *D. melanogaster*. Distribution of Epithelial Cell (D), Glia cell (E) and Neurons(F) in the t‐SNE MAP and their genes expression level in D’–F’. The marker genes corresponding to merged cell types and *D. melanogaster* marker genes are summarized in Data S4 and S5, respectively.

These cross‐species transcriptomic similarities at the cell‐population level support cell‐type annotation in *B. dorsalis* and suggest broadly comparable molecular features between corresponding antennal cell populations of *B. dorsalis* and *D. melanogaster*. For instance, *Obp19d* in epithelial cells likely serves as an odor buffer, clearing and isolating excess odorant molecules to protect olfactory neurons [45]. Similarly, *Hth*, a key determinant gene for antennal development causes antenna‐to‐leg transformation when its function is impaired [46] (Figure 2D,D′). In glial cells, *Ncc69* acts as a Na‐K‐Cl transporter, where it functions to maintain extracellular fluid and osmotic balance, thereby preventing fluid accumulation that would otherwise interfere with neural function [47]. *Drgx* regulates the development of motion‐sensitive T4/T5 neurons in *Drosophila* [48] (Figure 2E,E′). In antennal neurons, *Para* encodes the alpha subunit of voltage‐gated sodium channels, which are essential for sodium‐mediated action potentials and facilitate signal transmission, such as the propagation of mechanical signals in the *Drosophila* antenna [49]. *Rdl* encodes a ligand‐gated chloride channel that mediates the inhibitory effect of GABA on neuronal signaling and plays a critical role in odor coding [50] (Figure 2F,F′). In summary, our comparative analysis showed that major non‐neuronal structural and support‐cell populations, such as epithelial, support‐cell, glial, muscular, and hemocytic populations—displayed relatively high cross‐species mapping similarity between *B. dorsalis* and *D. melanogaster*. By contrast, although neuronal populations could be broadly matched at the major cell‐type level, their finer subcluster‐level correspondences were weaker (Figure S5A,B). As an additional reference, we also compared the *B. dorsalis* antennal atlas with a publicly available mosquito antennal single‐cell atlas. This comparison showed a broadly similar cell‐type‐dependent pattern of transcriptomic correspondence, in which candidate epithelial and support‐cell populations displayed stronger mapping than neuronal populations (Figure S6A,B). This disparity aligns with the distinct ecological niches of these dipteran species, suggesting that antennal neurons in *B. dorsalis* have evolved a unique gene regulatory network to adapt to environmental challenges specific to this species.

### 2.3 Neuronal Subclusters in *B. dorsalis* Reveal Olfactory Functional Diversity

To investigate the functional diversification of neuronal cells in the antennae of *B. dorsalis*, we performed a sub‐clustering analysis. The results demonstrated that high‐resolution clustering of antennal neurons identified 31 distinct sub‐clusters. The majority of these sub‐clusters were characterized by marker genes for chemosensory receptors, notably ionotropic receptors (IRs) and olfactory receptors (ORs) (Figure 3A,B). Interestingly, we found that all olfactory receptors in *B. dorsalis* with reported functions show independent distribution patterns in this atlas (Figure 3C). For example, olfactory receptors associated with methyl eugenol (ME) perception and behavioral response, *Or94b1*, *Or88as*, and *Or63a‐2‐v1* are expressed in three distinct cell clusters, implicating the involvement of three different olfactory neuron types in the detection of this compound by *B. dorsalis* [35, 51]. Others like *Or74a* as 1‐Nonanol in vitro ligand [52], *Or13a‐1* required for 3‐ol mediated oviposition [53], *Or43a‐1* required for benzo mediated oviposition [54], *Or10a* as ethyl benzoate in vitro ligand [55] and *Or94b‐2* as eugenol in vitro ligand [56]. These results suggest dominant receptor‐transcript signatures in ORN subclusters, broadly aligning with the conventional “one receptor‐one neuron” organization described in *D. melanogaster* [57]. In addition to the “one receptor‐one neuron” principle, recent studies showed that some olfactory neurons can co‐express multiple receptors [20, 58]. We found that receptor‐transcript co‐detection was also present in snRNA‐seq‐defined ORN subclusters of *B. dorsalis* (Figure 3D). For instance, transcripts from six olfactory receptor genes (*Or63a‐2‐v1*, *Or63a‐2‐v2*, *Or63a‐2‐v3*, *Or63a‐3*, *Or63a‐4‐v1*, and *Or63a‐4‐v2*) were co‐detected in adult ORN19, while transcripts from three Or67d receptor genes (*Or67d‐1*, *Or67d‐2*, and *Or67d‐3*) were co‐detected in the distinct adult ORN6 subcluster (Figure 3E). Pairwise Pearson correlation analysis of normalized expression values further showed positive correlations among the six Or63a receptor and three Or67d receptor transcripts (r = 0.12–0.79; Figure S7), supporting the robustness of this receptor‐transcript co‐detection pattern. Our previous genomic analysis of *B. dorsalis* revealed that some olfactory receptor genes have expanded through tandem duplication [42]. Here, we found that transcripts from some of these OR genes were co‐detected within the same ORN subclusters, suggesting that their expression regulation may not yet be fully differentiated after recent gene duplication.

**FIGURE 3:**
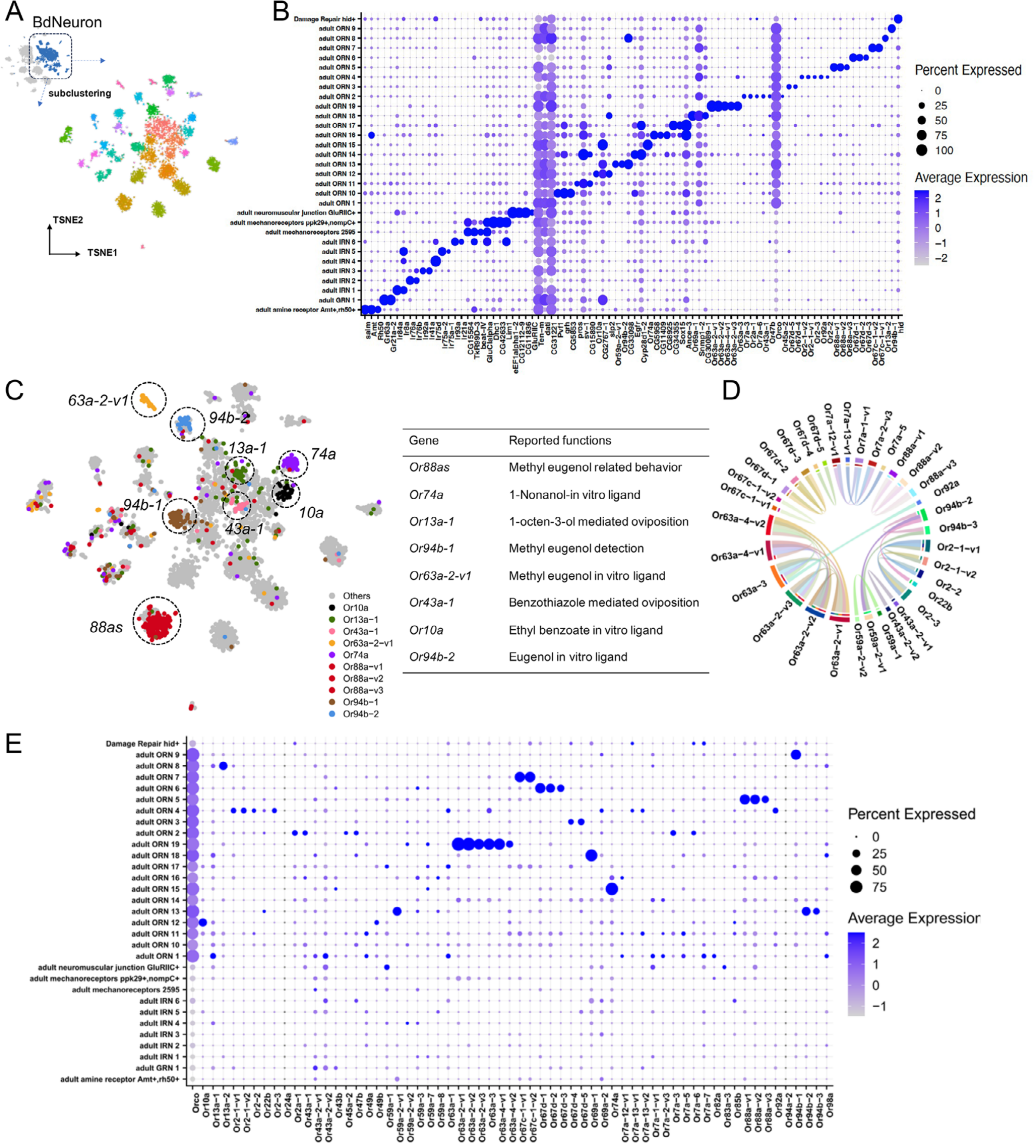
Subclustering analysis of neuronal cells. (A) A single t‐SNE map of the antenna neuron cells contains 31 distinct cell clusters that were annotated on the t‐SNE map. (B) Expression levels and the percentage of cells expressing marker genes in each neuronal subcluster are shown in a dot plot. The marker genes corresponding to neuronal subclusters are summarized in Data S7. (C) Distribution of currently characterized olfactory receptors in the neuronal cells t‐SNE map and their ligands listed in the table. (D) Chord plot showing co‐detected odorant receptor pairs: both genes are detected in over 10 cells, each with a normalized UMI count of at least 2. Receptor pairs meeting the predefined expression criteria and exhibiting a Pearson correlation coefficient of |r| > 0.1 across all cells are connected. (E) Expression levels and the percentage of cells expressing all olfactory receptor (OR) genes in each neuronal subcluster are shown in a dot plot.

Olfactory neurons represent the dominant population among antennal neuronal cells. These cells, which utilize the Orco protein for their primary role in chemosensory signal transduction, are also the most numerous cell type throughout the antenna (Figure 4A). Through a comparative cross‐species analysis as mentioned above, we aligned the single‐nucleus transcriptomes of antennal neuron cells between *B. dorsalis* and *D. melanogaster* (Figure S4B). Given the limited annotation in the *Drosophila* antennal cell atlas, most annotated ORN clusters in *B. dorsalis* mapped predominantly to unannotated *Drosophila* clusters (Figure 4B). We therefore treated these mappings cautiously and did not interpret the lack of *Drosophila* annotation alone as evidence of evolutionary divergence. To reduce the potential influence of incomplete annotation, we further examined annotated *D. melanogaster* ORN populations together with receptor orthology information. Several annotated *Drosophila* ORNs lacked clear *B. dorsalis* counterparts despite having recognizable receptor orthologs in *B. dorsalis*, whereas several mapped ORN pairs did not preserve orthologous receptor identity (Data S10). These results suggest that weaker ORN correspondence cannot be explained solely by incomplete *Drosophila* annotation and is consistent with cell‐type transcriptomic divergence and differences in receptor deployment among ORN populations. Consistently, these neuronal subclusters showed lower cross‐species transcriptomic similarity observed at the total cell cluster level (Figure S5A,C). Only a limited number of neuronal subclusters showed relatively high mapping scores, suggesting relatively strong cell‐type transcriptomic similarity between *B. dorsalis* and *D. melanogaster*. These included Johnston's organ neurons (JNs) and the carbon dioxide‐sensing *BdorGr63a* cell cluster (Figure S5D–F), consistent with potentially similar cellular roles across the two species. Some receptors (e.g., *Or88a*, *Or67d*) exhibit entirely different distributions in the neuronal cells of *B. dorsalis* and *D. melanogaster* (Figure S5G,H). Additionally, the ammonia‐sensitive *Amt* is found in two cell clusters in *B. dorsalis* compared to only one in *Drosophila* (Figure 4C,F), suggesting divergent ammonia‐sensing mechanisms between the two species.

**FIGURE 4:**
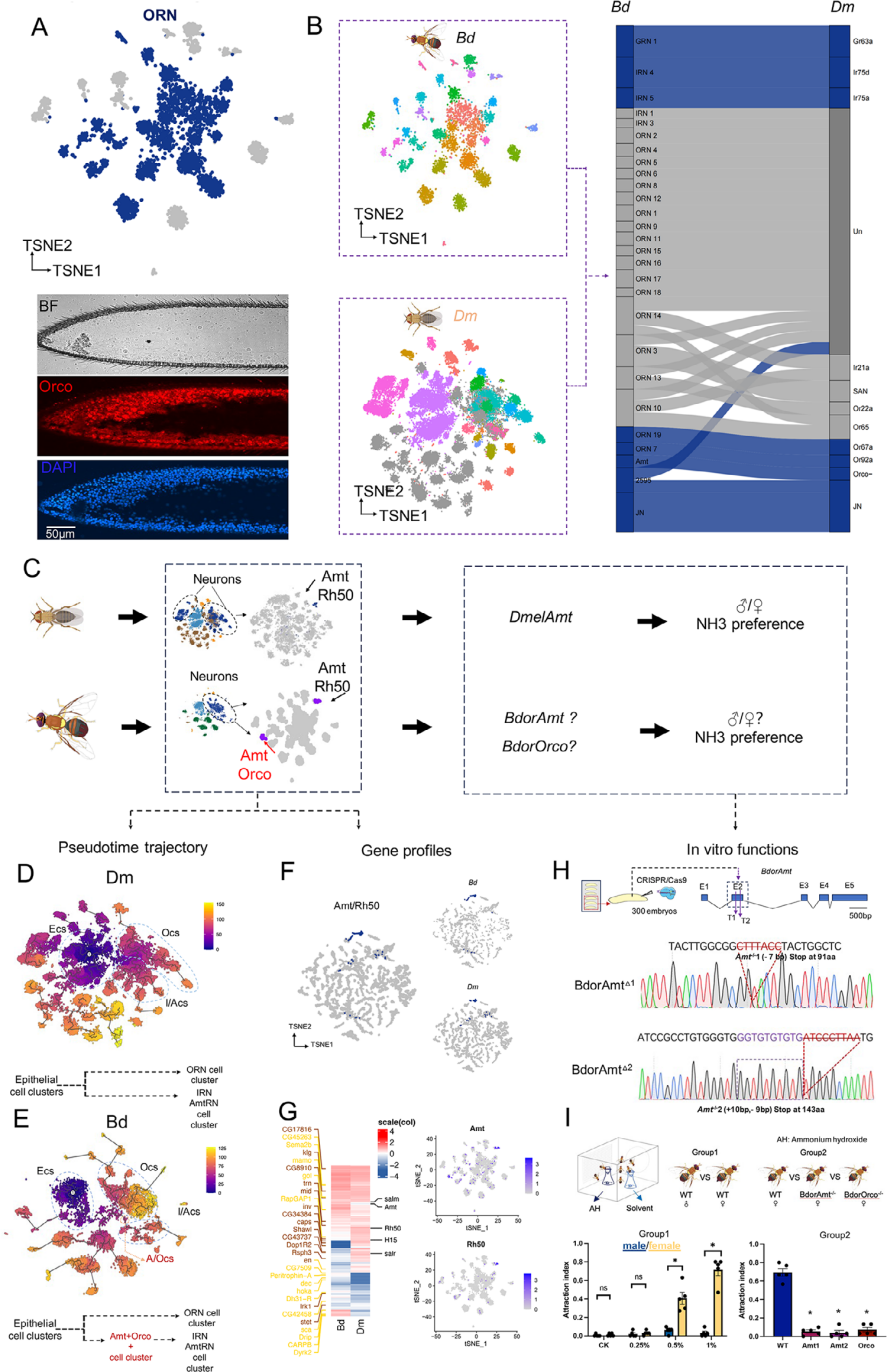
Cross‐species analysis of *B. dorsalis* and *D. melanogaster* neuronal cells. (A) Spatial expression pattern of olfactory neurons in the antenna. Orco protein was stained with the red color and DAPI stains nuclei. (B) Sankey plot summarizing the neuronal cell‐type mappings. Edges with alignment scores < 0.2 were omitted. For clarity, abbreviated cell‐type names are shown in the Sankey plot, with the corresponding full names provided in Data S6. (C) Comparison of the development, transcription, and potential functions of *Amt*‐related cell populations associated with ammonia (NH_3_) preference in *D. melanogaster* and *B. dorsalis*. (D,E) Pseudotime analysis of the trajectory of the principal cell populations in the antennae of *D. melanogaster* and *B. dorsalis*. Different colors represent the relative timing of the emergence of cell populations. The lower panel illustrates the developmental trajectory from epithelial cells to major olfactory neurons, highlighting that *B. dorsalis* exhibits a transcript co‐detection cell population of *Amt* and *Orco* prior to the formation of olfactory receptor neurons. (F) Distribution of *Amt* cells in the t‐SNE plot, showing the gene expression characteristics of *Amt* and *Rh50* in *D. melanogaster* and *B. dorsalis*. The gray area represents the overall cell population, and the marked blue dots indicate the locations of *Amt* cells. (G) Comparison of gene transcription expression within the *Amt* and *Rh50* cell populations between *D. melanogaster* and *B. dorsalis*. The marked genes are the top 20 marker genes, with black representing conserved genes, yellow (*B. dorsalis*) and brown (*D. melanogaster*) indicating different genes. On the right, representative marker genes show the annotated cell populations of *Amt* and *Rh50* in the neural cell groups of the *B. dorsalis*. (H) Establishment of the *BdorAmt* knockout strain in *B. dorsalis* using CRISPR/Cas9. Two mutant types were obtained: the *BdorAmt* ^△^1 mutant has a 7 bp deletion, predicted to result in a stop codon at the 91st amino acid; the *BdorAmt* ^△^2 mutant has a 10 bp insertion and a 9 bp deletion, predicted to result in a stop codon at the 143rd amino acid. (I) Behavioral assay of the attractiveness of ammonia to *B. dorsalis*. Group 1: Comparison of the attraction index (mean ± SEM) of wild‐type (WT) male and female flies to different concentrations of ammonia hydroxide (0.25%, 0.5%, 1%). Wilcoxon rank‐sum tests were used for female–male comparisons at each concentration; n = 5, * indicates *p* < 0.05, and ns indicates no significance. Group 2: Comparison of the attraction ability (mean ± SEM) to 1% ammonia hydroxide between wild‐type (WT) and three mutants (*BdorAmt* ^△^1, 2 and *BdorOrco* ^−/−^), with WT to mutant are shown. Pairwise Wilcoxon rank‐sum tests were used, with *p* values adjusted for multiple comparisons using the Benjamini–Hochberg method; n = 5, * indicates *p* < 0.05.

### 2.4 Amt‐Expressing Neurons Mediate Ammonia Perception in an Orco‐Dependent Manner

Since the *Amt* cluster in *B. dorsalis* has been found to be syntenic with that in *D. melanogaster*, but with lower similarity (Figure S5D), we focus on this *Amt‐*associated candidate neuronal populations for further analysis to verify its potential function in ecological adaptation. In *D. melanogaster*, ammonia‐responsive ORNs co‐express two members of the *Amt/MEP/Rh* ammonium‐transporter superfamily, *Amt* and *Rh50*. *Amt* functions as a non‐canonical olfactory receptor required for ammonia detection, and relatively low concentrations of ammonia attract both male and female flies [59]. In the antennal atlas of *B. dorsalis*, we identified a candidate neuronal population characterized by *BdorAmt* and *BdorRh50* transcript co‐detection, which was further supported by HCR RNA FISH (Figure S8A–C). This finding is consistent with partial conservation of the molecular identity of the *Amt/Rh50*‐expressing ammonia‐sensing ORNs described in *D. melanogaster*. We also identified an additional candidate population showing *BdorAmt* and *BdorOrco* transcript co‐detection. This candidate population was further supported by targeted computational analyses after ambient RNA correction, including its retention in ORN16, significant enrichment relative to other ORN populations, and detection in both female and male antennal libraries (Figure S8D–F), suggesting potential species‐associated diversification of *Amt*‐related candidate neuronal populations. Ammonia is one of the key components that attract tephritids to protein bait [60, 61]. However, it remained unknown whether low concentrations of ammonia attract both male and female *B. dorsalis*, as they do in *D. melanogaster*, and whether *BdorAmt* is required for this behavioral response (Figure 4C).

Although corresponding *Amt*‐related cell populations were identified in both *D. melanogaster* and *B. dorsalis*, cell clustering and cross‐species mapping scores alone were insufficient to characterize their differences or infer their potential regulatory roles. To further compare the corresponding *Amt*‐related populations between the two species, we examined two complementary features: their positions within pseudotime‐inferred antennal cell‐state trajectories and their cluster‐enriched marker‐gene profiles. For the first analysis, pseudotime analysis in *D. melanogaster*, with epithelial cells designated as the trajectory root, resolved two principal branches extending toward support‐cell and neuronal states (Figure 4D). This topology is consistent with the canonical sensory organ lineage in *Drosophila*, in which sensory organ precursors generate *pIIa* and *pIIb* daughter cells that subsequently give rise to support cells and neurons, respectively [62, 63]. A broadly similar branching topology was inferred in *B. dorsalis*, with epithelial‐associated states positioned at the root and support‐cell and neuronal states distributed along separate branches. The inferred *B. dorsalis* pseudotime ordering was robust to cell subsampling: across 100 iterations using random 80% cell subsets, pseudotime values remained correlated with those from the complete dataset (mean Spearman's ρ = 0.76 ± 0.05; range, 0.69–0.87; Figure S9). Notably, candidate cells characterized by *BdorAmt* and *BdorOrco* transcript co‐detection occupied an intermediate position within the adult pseudotime‐inferred antennal cell‐state trajectory, near ORN and *IR/Amt*‐associated neuronal states (Figure 4E). This pattern suggests an overlap between *BdorAmt* and *BdorOrco* transcription within the adult pseudotime‐inferred cell‐state continuum, which may be associated with the molecular identity or function of ammonia‐associated neurons. For the second analysis, we independently compared the cluster‐enriched marker‐gene profiles of the corresponding *Amt/Rh50*‐associated populations in *B. dorsalis* and *D. melanogaster* (Figure 4F). For each population, the 20 positively enriched marker genes with the smallest adjusted *p* values were selected. *Amt*, *Rh50*, *salm*, *salr*, and *H15* were shared among the top‐ranked markers of both populations, consistent with partial conservation of their molecular identities. In contrast, the other 15 top‐ranked markers did not overlap between the two species. For example, *Dop1R2* was specifically enriched in *D. melanogaster*, whereas *Dh31R* was specifically enriched in *B. dorsalis*, highlighting species‐associated differences in their transcriptional programs (Figure 4G).

Given these molecular differences between the corresponding *Amt*‐related cell populations in *D. melanogaster* and *B. dorsalis*, we next asked whether they are reflected in species‐specific behavioral responses to ammonia and in the underlying receptor dependence. To test the behavioral relevance of ammonia, we first performed olfactory trap assays using ammonium hydroxide (Figure 4I). The results showed that low doses of ammonia (0.5% and 1.0%) attracted females, but not males, in *B. dorsalis*, in contrast to *D. melanogaster* (Figure 4I, Group 1). Interestingly, within the *BdorAmt*/*BdorRh50* transcript*‐co‐*detected candidate cells in the *Amt* cluster, *BdorAmt* transcript levels tended to be higher in females than in males (Figure S10A). To further examine the role of *BdorAmt* in this response, we generated *BdorAmt* knockout mutants using CRISPR/Cas9. Following embryo injection, we obtained two mutant lines: *BdorAmt*Δ1 carried a 7‐bp deletion, whereas *BdorAmt*Δ2 carried a 10‐bp insertion together with a 9‐bp deletion. These mutations were predicted to cause premature truncation of the protein at amino acid positions 91 and 143, respectively (Figure 4H). Female mutants were then subjected to olfactory trap assays using 1% ammonium hydroxide as the odor source. The attraction to ammonia was nearly abolished in *BdorAmt* mutant females compared with wild‐type flies, supporting a critical role for *BdorAmt* in female attraction to ammonia (Figure 4I, Group 2). Because we also identified cells co‐expressing *BdorOrco* and *BdorAmt*, we next examined the behavioral response to ammonia in female from a previously generated and validated *BdorOrco* knockout line [35]. Interestingly, *BdorOrco* mutant females also showed a near‐complete loss of attraction to low doses of ammonia, again differing from the situation in *D. melanogaster* (Figure 4I, Group 2).

### 2.5 The Derived Ammonia‐Sensing Logic Is Tightly Linked to the Specific Ecological Demands of *B. dorsalis*

Under natural conditions, researchers have consistently observed tephritids feeding on bird droppings [6, 8, 9, 10], which are a natural source of ammonia [64]. Therefore, we anticipate that ammonia may serve as a cue to help these flies locate this potential nutrient source by guiding their foraging behavior. We applied Nessler's reagent spectrophotometry to compare the ammonia release rates between bird droppings and 0.5%–2% ammonia hydroxide. The results indicated that 5 g of bird droppings is able to release comparable amounts of ammonia to that of 1% ammonia hydroxide, which actively traps female adults (Figure 5A). Consequently, we used the bird droppings in the above amounts to perform another olfactory trap assay to compare their attractiveness between male and female adults (Figure 5B). The results indicated that, consistent with 1% ammonia hydroxide, only female adults could be trapped by the bird droppings (Figure 5B, Group 3). To further clarify the relationship between *BdorAmt* and female behavior in locating ammonia from bird droppings, we compared the attraction of wild‐type and mutant females to this nutrient source in the olfactory trap assays. The results were largely consistent with the findings from the above experiment; the behavior of females toward bird droppings was drastically diminished when *BdorAmt* was knocked out. Meanwhile, the behavioral patterns were also similar when testing the *BdorOrco* mutant's attraction to bird droppings (Figure 5B, Group 4). These results support the notion that low dose of ammonia as an important bird‐dropping‐associated cue that preferentially attracts females and involves both *BdorAmt* and *BdorOrco*.

**FIGURE 5:**
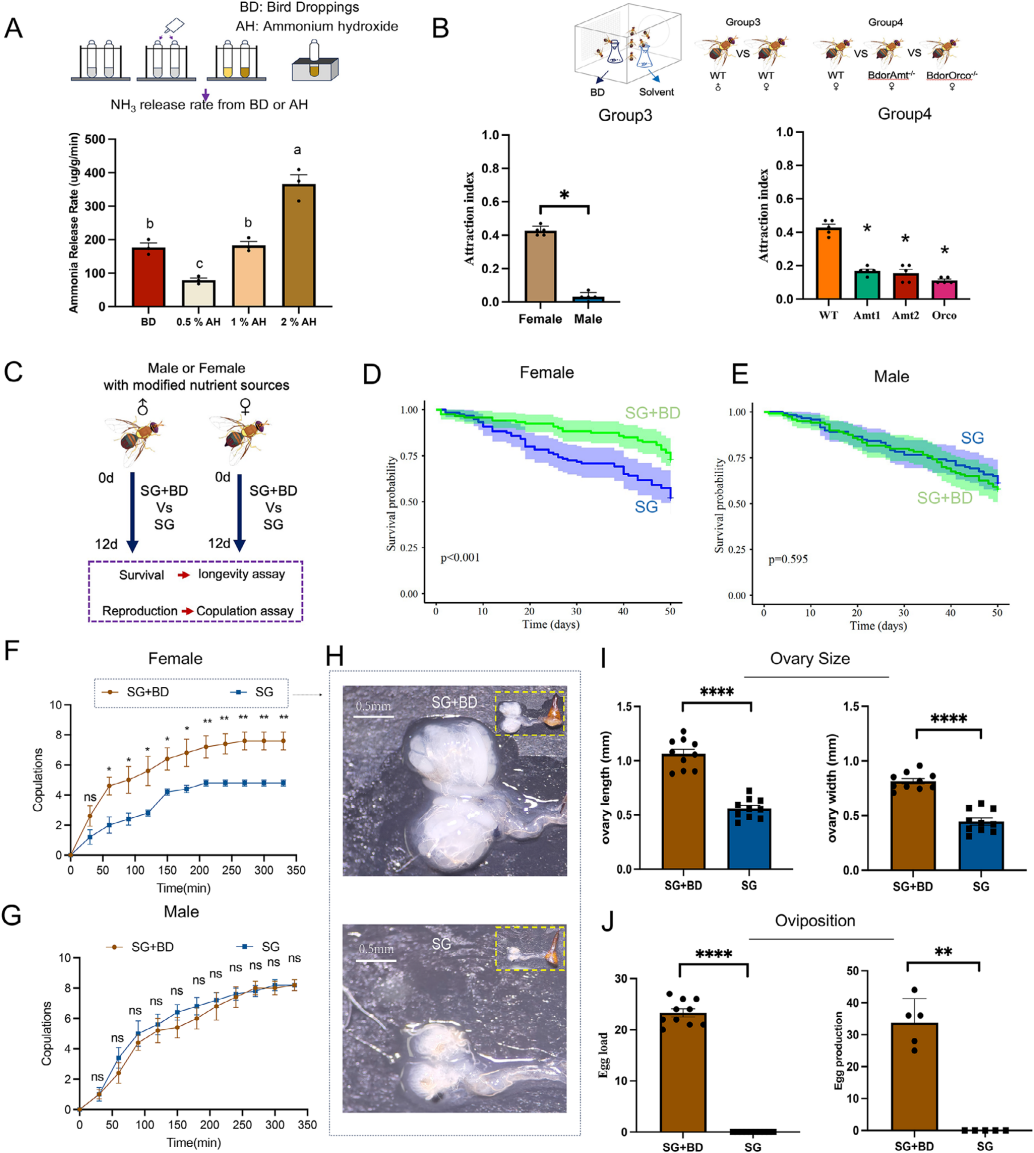
The roles of *BdorAmt* and *BdorOrco* in ammonia‐mediated behaviors. (A) Ammonia release from natural sources (bird droppings) quantified (mean ± SEM) using the Nessler's Reagent Spectrophotometry, with n = 3 biological replicates, each consisting of 10 mL of absorbing solution. Statistical test was performed by One‐Way ANOVA (followed by Tukey's test, normally distributed data). Different letter denotes significant differences (α = 0.05). (B) Behavioral responses of adult *B. dorsalis* to bird droppings, assessed using olfactory trap assays. Group 3: Differences in behavioral responses (mean ± SEM) between male and female adults toward bird droppings, with n = 5 biological replicates, each containing 30 individuals. Statistical test was performed by Wilcoxon rank‐sum tests. * indicate *p* < 0.05; Group 4: Effects of knocking out *BdorAmt* and *BdorOrco* on the behavioral responses (mean ± SEM) of females to bird droppings, with WT to mutant are shown. n = 5 biological replicates, each containing 30 individuals. Statistical test was performed by Pairwise Wilcoxon rank‐sum tests, with *p* values adjusted for multiple comparisons using the Benjamini–Hochberg method. * indicate *p* < 0.05. (C) Experimental design for assessing survival and reproductive fitness of adults with bird droppings as an additional nutritional source. (D‐E) Female: Survival curve of females when provided with bird droppings as an additional nutritional source; Male: Survival curve of males under the same conditions. Shaded areas indicate 95% confidence intervals. With n = 3 biological replicates, each containing 40 individuals. Statistical test was performed by Kaplan–Meier survival analysis with 95% confidence intervals, with the log‐rank test applied to compare the survival curves of treated adults against those of untreated controls (α = 0.05). (F,G) Changes in numbers of copulations (mean ± SEM) in adults with bird droppings as an additional nutritional source. Female: Only females consuming bird droppings; Male: Only males consuming bird droppings. With n = 5 biological replicates, each containing 20 individuals (♂:♀ = 1:1). Statistical test was performed by two‐sided unpaired *t*‐test (normally distributed data) or Mann‐Whitney U test (non‐normally distributed data). ns denotes *p*>0.05, *p < 0.05, **p < 0.01. (H) Morphology of the ovary under conditions where bird droppings serve as an additional nutrient source. (I) Effects of this nutrient source on the length and width (mean ± SEM) of the ovary. With n = 10 biological replicates, each replicate represents a single ovary. Statistical test was performed by two‐sided unpaired *t*‐test (normally distributed data) or Mann‐Whitney U test (non‐normally distributed data). ****p < 0.0001. (J) Impact on egg load and production (mean ± SEM) associated with the additional nutrient source. For egg load, n = 10 biological replicates were used, and data for each replicate were derived from a single ovary. For oviposition, n = 5 biological replicates were analyzed, with each replicate representing the total egg production of 10 females. Statistical test was performed by two‐sided unpaired *t*‐test (normally distributed data) or Mann‐Whitney U test (non‐normally distributed data). **p < 0.01, ****p < 0.0001.

Ammonia is released during the decomposition of protein‐containing organic materials and appears to be important for the survival of various species [65]. Since only female *B. dorsalis* are attracted to bird droppings, and mated females showed significantly reduced attraction to NH3 compared with unmated females (Figure S10B), we hypothesized that this natural resource may provide supplementary nutritional benefits linked to female survival and reproductive state. Therefore, we established two groups: the treatment group, which was provided with bird droppings and sugar as food for both male and female adults, and the control group, which received sugar only. These two groups of males and females were subsequently used to perform survival and reproduction assays (Figure 5C). The survival curve indicated that with bird droppings as an additional nutrient, the female survival rate was significantly higher (*p* < 0.001; Figure 5D, Female). In contrast, the survival rate of males did not differ significantly with or without bird droppings (p = 0.595; Figure 5E, Male). A consistent pattern was also observed in copulation behavior, with a significantly decreased mating success in females when bird droppings were not available as additional nutrients (Figure 5F, Female). However, the mating rate of males was not affected regardless of whether bird droppings were provided (Figure 5G, Male). Since the ovarian development of female tephritids is highly dependent on protein intake, probably with the specific “protein hunger” [64], we subsequently dissected the ovaries from those females that had been prepared in the two groups, with or without the treatment of bird droppings. The results indicated that when bird droppings were provided, the ovaries in females significantly enlarged (Figure 5H,I) and contained morphologically intact eggs, and these eggs were finally oviposited (Figure 5J). However, these eggs failed to hatchcou, which indicated that female insects still require key nutrients from other sources to complete embryo development and achieve successful reproduction. Taken together, these results suggest that ammonia‐associated attraction to bird droppings involves *BdorAmt*‐associated candidate neuronal populations and contribute to female survival and reproductive‐associated traits. These findings further support the roles of neuronal clusters in the ecological adaptation to their environment.

## 3 Discussion

Uneven transcriptomic divergence across cell types has been reported in Caenorhabditis, with neuronal populations exhibiting greater transcriptomic divergence than several non‐neuronal cell types [66]. Our study extends comparative evidence for this pattern to an insect peripheral olfactory organ, suggesting that cell‐type‐dependent transcriptomic divergence may occur across diverse animal lineages. This pattern may represent one route through which insect olfactory systems adapt to distinct ecological niches without necessarily requiring full‐scale reorganization of antennal cell‐type composition. In *B. dorsalis*, this divergence is associated with a distinct ammonia‐response pathway in which female attraction to ammonia requires both *BdorAmt* and *BdorOrco*, contrasting with the *Orco*‐independent *Amt*‐mediated response in *D. melanogaster*. This difference, together with our feeding experiments showing that access to bird droppings improved adult longevity, mating success, and ovarian development, illustrates how cell‐type‐dependent transcriptomic divergence may contribute to species‐specific ecological adaptation.

Consistent with this view, most structural cell types showed strong cross‐species correspondence between *D. melanogaster* and *B. dorsalis*. Epithelial, support, and glial cells are core components of the sensillum, the basic functional unit of the insect antenna [67, 68], whereas muscle cells and hemocytes contribute to antennal movement, maintenance, and tissue homeostasis [69, 70]. The overall conservation of these cell populations suggests that core antennal functions are maintained across these dipteran species. A limited number of neuronal populations also exhibited conservation, including candidate CO_2_‐responsive neurons marked by *Gr21a* and *Gr63a* orthologs [71, 72], as well as Johnston's organ neurons expressing Dhc93AB [38]. In contrast, most sensory neuronal populations showed weaker correspondence and greater transcriptional divergence between species. Notably, substantial differences were observed even among neurons expressing orthologous olfactory receptors, suggesting that ecological adaptation may rely less on large‐scale changes in major antennal cell‐type composition and more on cell‐type‐dependent transcriptomic divergence among olfactory sensory neuron populations, including changes in receptor deployment and ammonia‐response pathways.

Ammonia sensing provides a particularly illustrative example of this principle. In *Drosophila*, *Amt* is expressed both in support cells associated with coeloconic sensilla [73] and in ac1 olfactory neurons, where it functions as an ammonia receptor [59]. In *B. dorsalis*, we similarly detected *BdorAmt* expression in support cell clusters and identified a neuronal subtype co‐expressing *BdorAmt* and *BdorRh50*, supporting partial conservation of the ammonia‐sensing system across Diptera. Our olfactory trap assays further demonstrated that *BdorAmt* is required for attraction to ammonia. However, unlike in *Drosophila*, where ammonia is attractive to both sexes, ammonia preferentially attracted female *B. dorsalis*, suggesting that this cue has acquired a more specific ecological role in this species. Given that females use ammonia as an important cue to locate bird droppings, and bird‐dropping supplementation improved female survival and reproductive‐associated traits under laboratory conditions, this behavioral shift may reflect an association between ammonia‐guided foraging and female‐biased nutritional demands. At the same time, *BdorAmt/BdorRh50* transcript*‐co‐*detected candidate cells were detected in both sexes, and *BdorAmt* transcript levels in this population tended to be higher in females than in males. These observations suggest that the sex difference in ammonia attraction may be shaped by both peripheral *BdorAmt* transcript‐level differences and downstream olfactory processing in the brain, rather than by the peripheral presence or absence of this neuronal population alone.

A second, and potentially derived, feature of the *B. dorsalis* antenna is the presence of a distinct neuronal cluster co‐expressing *BdorOrco* and *BdorAmt*. This configuration differs from that of *D. melanogaster*, in which loss of *DmOrco* does not abolish adult attraction to ammonia [65]. By contrast, disruption of *BdorOrco* in our study largely phenocopied the *BdorAmt* mutant, suggesting that ammonia sensing in *B. dorsalis* depends on a distinct ammonia‐response pathway involving both genes. How *BdorOrco* contributes to *BdorAmt*‐dependent sensing remains unclear. Our trajectory analysis suggests that *BdorOrco* is more likely to influence ammonia sensing indirectly by affecting the developmental trajectory, maturation, or physiological state of *Amt*‐associated neuronal populations, rather than acting solely as an acute signaling component. However, this developmental model requires further validation. First, stage‐resolved in situ hybridization should confirm the existence and dynamics of *Amt/Orco* co‐expressing cells during early antennal development. Second, developmental single‐cell transcriptomics should reconstruct the transcriptional networks of *Amt/Orco*‐ and *Amt/Rh50*‐associated populations and define their relationship during normal antennal development. Third, functional assays in *BdorOrco* mutants, such as single‐sensillum recording combined with cell‐type identification, should determine whether ammonia responses of *Amt/Rh50*‐associated neurons are disrupted. Finally, single‐cell transcriptomics of *BdorOrco* mutant antennae should be compared with wild‐type developmental networks to test whether *Orco* loss shifts, arrests, or diverts *Amt/Rh50*‐expressing cells from their normal developmental trajectory.

Beyond these conceptual insights, our study also provides a foundational cellular resource for the *B. dorsalis* antenna. We generated an snRNA‐seq atlas comprising 30 major cell types and further resolved neuronal populations into 31 subtypes, enabling more precise annotation of chemosensory, support, and mechanosensory cell states. This atlas is broadly comparable to available antennal atlases from *D. melanogaster* and mosquitoes [19, 21], and it captures candidate cell populations relevant to key behavioral traits, including neurons associated with methyl eugenol response, CO_2_‐sensing, ammonia sensing, and Johnston's organ function [35, 38, 59, 71, 72]. In addition, the transcription factor regulatory resource inferred from *D. melanogaster* provides a useful starting point for future cell‐type‐specific regulatory analysis in *B. dorsalis*. Together, these resources establish a framework for functional annotation and for identifying molecular targets with potential value in pest management.

More broadly, this work illustrates how single‐cell and single‐nucleus approaches can be used to bridge model and non‐model insects in studies of adaptation. By comparing pest species with genetically and functionally tractable model systems such as *Drosophila*, it becomes possible to infer candidate cell identities, prioritize molecular targets, and test how sensory systems are reshaped in agricultural pests. Future efforts could further improve this framework by enriching specific cell populations through transgenic or in situ labeling strategies, such as PERFF‐seq [74], followed by targeted RNA‐seq or snRNA‐seq. In parallel, applying HCR‐ or SABER‐based RNA FISH to isolated antennal nuclei or dissociated antennal cells in *B. dorsalis* would be a useful approach for validating expression patterns that are difficult to resolve among densely packed cells in intact antennal tissue. Such approaches would increase the resolution of rare but behaviorally important cell types, including *BdorOr94b1*‐positive neurons or distinct *BdorAmt* populations, and help reveal the dynamic transcriptional programs underlying species‐specific sensory adaptations.

## 4 Materials and Methods

### 4.1 Insect Stocks

*B. dorsalis* laboratory lines were maintained at the Agricultural Genomics Institute at Shenzhen, Chinese Academy of Agricultural Sciences. Colonies were reared under a 14 h light/10 h dark photoperiod at 26 ± 1°C and 60 ± 5% relative humidity. Larvae were reared on an artificial diet consisting of banana, corn flour, yeast, sugar, and cellulose paper. Adults were provided with a mixture of brewer's yeast powder and granulated sugar. When larvae reached the third instar, they were transferred directly to small plastic cages (18 cm × 12.5 cm × 14 cm) for pupation. Adults were sexed at 3–4 days post‐eclosion, well before sexual maturity at approximately 9 days post‐eclosion, and maintained separately as unmated adults until 12 days of age for dissection. Antennae were dissected in Schneider's medium, transferred to 1.5 mL microcentrifuge tubes containing 100 µL of the same medium, and stored at −80°C until use. Wild type and gene‐edited lines were maintained under the same rearing conditions. The *BdorOrco* ^−/−^ line used in this study was previously generated and validated[35], whereas the *BdorAmt* mutant lines were generated in the present study using CRISPR/Cas9 as described in the following section. All adults, larvae, pupae, and insect‐containing waste were inactivated before disposal according to institutional laboratory management procedures.

### 4.2 Single Nucleus Isolation and Sequencing

Antennal tissues of adult male and female *B. dorsalis* were dissociated into single nuclei following the method of Li et al. (2022) [19] with minor modifications. All male and female individuals used for snRNA‐seq were unmated. Antennae were dissected from wild‐type adults of 12 days under a microscope and immediately transferred into 1.5 mL tubes, with a 60 of individuals per sample. Samples were rapidly frozen in liquid nitrogen to minimize exposure to room temperature and stored at −80°C until use. For nuclei preparation, frozen samples were thawed on ice and resuspended in 100 µL PBS containing 0.5% BSA and RNase inhibitor (Promega, N2615). After centrifugation at 800 g for 30 s at 4°C, the supernatant was removed and 1 mL lysis buffer (Data S12) was added. The lysate was transferred to a 2 mL Dounce homogenizer and gently homogenized with 30 strokes using a loose pestle followed by 40 strokes with a tight pestle to release nuclei. The homogenate was filtered through a 40 µm cell strainer and centrifuged at 800 g for 10 min at 4°C. After removing the supernatant, the pellet was resuspended in PBS containing 0.5% BSA and RNase inhibitor. The nuclei suspension was then passed through a 40 µm Flowmi strainer into new tubes. Nuclei were stained with Hoechst 33342 for more than 5 min and sorted by fluorescence‐activated cell sorting (FACS). Single nuclei were collected into tubes for subsequent 10×Genomics processing.

Single‐nucleus RNA sequencing libraries were prepared using the 10× Genomics Chromium Single Cell 3′ Reagent Kit v3 (Chromium, PN‐1000075) following the manufacturer’ s protocol. Nuclei suspension was loaded onto a Chromium Controller with a Single Cell Chip A to generate gel beads‐in‐emulsion (GEMs), each containing an individual nucleus with barcoded gel beads and reverse transcription reagents. Messenger RNA from each cell was reverse transcribed to complementary DNA carrying unique molecular barcodes. After GEM generation, emulsions were broken and cDNA was purified with DynaBeads MyOne Silane Beads (Thermofisher, 37002D), followed by PCR amplification and purification with SPRIselect magnetic beads. Amplified cDNA underwent fragmentation, end repair, A‐tailing, adaptor ligation, and index PCR to produce sequencing‐ready libraries. Library quality and fragment size were examined with an Agilent 2100 Bioanalyzer using the High Sensitivity DNA Kit (Agilent, 5067‐4626), and concentration was measured with a Qubit dsDNA HS Assay Kit (Thermofisher, Q328520). Libraries showing fragment sizes between 400 and 600 base pairs were sequenced on an Illumina NovaSeq 6000 or NextSeq 500 platform using paired‐end reads.

### 4.3 snRNA‐seq Dataset Processing

Raw sequencing data quality was assessed using FastQC. Raw sequencing reads were processed using the Cell Ranger (version 7.0.1) count pipeline (10× Genomics) to generate single‐nucleus gene expression matrices for each sample. Single‐nucleus analyses were performed using the Seurat package (version 5.4.0). Female and male *B. dorsalis* antennal datasets were merged to construct a unified cell atlas, allowing consistent identification and annotation of shared cell populations while enabling subsequent comparisons of sex‐specific gene expression within the same cellular framework. Batch effects between the two datasets were corrected using the Harmony package.

Raw gene expression counts were normalized using Seurat's LogNormalize method (NormalizeData, default parameters), in which gene counts for each cell were divided by the total UMI counts, multiplied by a scale factor of 10,000, and log‐transformed. The 2000 most highly variable genes were identified using FindVariableFeatures, followed by data scaling using ScaleData and principal component analysis (RunPCA). Harmony was applied to the PCA embeddings to correct batch effects between female and male samples.

Cell‐cell nearest‐neighbor graphs were constructed in the Harmony‐corrected low‐dimensional space using the first 40 Harmony components (FindNeighbors, reduction = “harmony,” dims = 1:40). Cell clustering was performed using the Louvain algorithm implemented in FindClusters, and two‐dimensional visualization was generated using both UMAP (RunUMAP) and t‐SNE (RunTSNE) based on the same Harmony embedding.

To evaluate clustering robustness, several complementary analyses were performed. Clustering stability across different resolution parameters (0.2, 0.3, 0.4, 0.5, 0.6, 0.8, and 1.0) was assessed using the clustree package to visualize the hierarchical relationships among clusters. The consistency between clustering algorithms was evaluated by comparing Louvain (algorithm = 1) and Leiden (algorithm = 4) clustering at a resolution of 0.6, and the similarity between the resulting cluster assignments was quantified using the Adjusted Rand Index (ARI) implemented in the mclust package. In addition, clustering analyses were repeated using the first 20, 30, and 40 Harmony components to assess the effect of dimensionality on clustering topology. The effectiveness of Harmony integration was further evaluated by visualizing the distribution of cells from different biological samples in the Harmony‐corrected embedding to confirm adequate batch mixing while preserving biologically meaningful cell‐type separation. Cell types were annotated according to canonical marker genes together with previously reported marker genes from insect antennal cell atlases.

Pairwise Pearson correlation coefficients were calculated across all cells using Seurat log‐normalized expression values. For receptor transcripts co‐detection analysis, odorant receptor pairs were included only when both genes were detected in more than 10 cells and each gene exhibited a normalized expression value of at least 2. Receptor pairs with an absolute Pearson correlation coefficient (|r|) greater than 0.1 were considered co‐expressed and were connected in the chord diagram, highlighting receptor pairs with consistent co‐detection patterns across neuronal cells.

### 4.4 Pseudotime Analysis

Pseudotime analysis was performed using Monocle3 (version 1.4.26). The annotated Seurat object was converted into a Monocle3 cell data set using as.cell_data_set. Data preprocessing was performed with preprocess_cds using the first 50 principal components (num_dim = 50), followed by cluster_cells to generate the partition information required by Monocle3. To maintain consistency with the Seurat analysis, the t‐SNE coordinates, cell‐type annotations, and cluster identities generated in Seurat were transferred to the Monocle3 object. All cells were assigned to a single partition, and trajectory inference was performed using learn_graph with use_partition = FALSE.

The developmental trajectory was rooted in the epithelial cell‐1 population, which was selected as the earliest developmental state based on the expression of epithelial marker genes and the absence of antennal stem cells. Pseudotime ordering was then computed using order_cells.

The robustness of the inferred trajectory was evaluated by downsampling analysis. In each of 100 independent iterations, 80% of cells were randomly sampled from the original dataset, and the complete trajectory inference procedure, including preprocessing, clustering, graph learning, and pseudotime ordering, was repeated using the same parameters as those applied to the full dataset. Spearman's rank correlation coefficient was calculated between the pseudotime values of cells shared by the downsampled and full datasets. The average correlation coefficient (Spearman's ρ = 0.76 ± 0.05) indicated that pseudotime ordering was highly reproducible and robust to cell subsampling.

### 4.5 Cross‐Species Analysis by Samap

Cross‐species analysis was performed using SAMap. The datasets included the *B. dorsalis* antennal single‐cell dataset generated in this study and the published *D. melanogaster* and *Aedes aegypti* antennal single‐cell dataset obtained from the SCOPE database (https://flycellatlas.org/, https://cells.ucsc.edu/?ds = mosquito). Orthologous gene relationships between the two species were used to construct the homology graph for cross‐species alignment. Each dataset was first processed independently using the Self‐Assembling Manifold (SAM) algorithm to construct species‐specific manifolds, followed by manifold alignment using SAMap.

Cell‐type correspondence between the two species was quantified using the get_mapping_scores function in SAMap, which calculates mapping (alignment) scores between annotated cell types based on the aligned manifolds. For downstream analyses, only cell‐type pairs with mapping scores greater than 0.2 were considered conserved. The resulting mapping score matrix was exported and visualized in R, where Sankey diagrams were generated to illustrate the correspondence between cell types of the two species. In the Sankey diagrams, each node represents a cell type and connecting lines indicate the strength of cross‐species mapping relationships. To further evaluate the transcriptional conservation of aligned cell types, the top 100 marker genes from each cluster in both species were used to calculate average expression levels, which were visualized as heatmaps in R.

### 4.6 Transcription Factors Enrichment and SCENIC Analysis

Transcription factor regulatory network analysis was performed using SCENIC (version 1.1.2‐01) in R (version 4.3.2). The input gene expression matrix was obtained from Seurat‐processed single‐cell transcriptomic data. SCENIC was initialized using the *D. melanogaster* cisTarget database (dm6‐5kb‐upstream‐full‐tx‐11species.mc8nr.genes_vs_motifs). Prior to network inference, genes were filtered using the geneFiltering function with minCountsPerGene = 3 × 0.01 × nCells and minSamples = 0.001 × nCells, resulting in 3821 genes retained for downstream analysis. Gene regulatory networks were inferred using the GRNBoost2 algorithm implemented in the Arboreto Python package, with a random seed of 2023L and transcription factors obtained from the cisTarget database. The resulting adjacency matrix was subsequently imported into the SCENIC workflow for regulon identification by RcisTarget motif enrichment analysis. Only regulons containing at least 10 target genes were retained. Regulon activity in individual nucleus was quantified using AUCell, with aucMaxRank set to the top 10% of ranked genes. Unless otherwise stated, all remaining parameters were set to their default values.

### 4.7 Immunostaining and Confocal Microscopy

Adult female and male *B. dorsalis* aged 7–10 days were dissected and anesthetized with CO_2_ to obtain the antennae tissues under a stereomicroscope. The antennae were embedded in optimal cutting temperature compound (OCT), oriented transversely, and rapidly frozen in liquid nitrogen. Cryosections of 10–20 µm thickness were prepared using a cryostat set to −25°C, placed onto poly‐L‐lysine‐coated slides, and stored at 4°C before staining. The following steps were performed at room temperature unless otherwise noted. Slides were fixed with 4% formaldehyde dissolved in 1× PBST (1× PBS + 0.3% Triton X‐100) for 1 h. After fixation, the slides were subsequently treated with blocking buffer (1× PBS + 0.3% Triton X‐100 + 5% NDS (normal donkey serum)) for 1 h. Following the blocking step, the primary antibody (custom‐made reagent made by Sangon Biotec, Shanghai, China) was added in the working solution (1× PBS + 0.3% Triton X‐100 + 1% BSA) at a dilution ratio of 1:1000, and the samples were incubated overnight at 4°C. After this, the samples were washed three times for 5 min each in PBST. They were then incubated with the secondary antibody (Donkey Anti‐Rabbit IgG H&L (Alexa Fluor 555)) at a dilution rate of 1:1000 in the dark. The sections were washed three times for 5 min each in PBST and finally covered with mounting medium (Beyotime, P0133, containing Hoechst 33342) and sealed with nail polish. The samples were stored at 4°C and imaged using a Nikon Eclipse Ti2 inverted microscope system.

### 4.8 HCR RNA FISH Validation

Adult female and male *B. dorsalis* aged 7–10 days were dissected and anesthetized with CO_2_ to obtain the antennae tissues under a stereomicroscope, immediately embedded in OCT compound, and sectioned into 14‐µm slices using a Leica CM1950 cryostat. The resulting cryosections were fixed for 30 min at 4°C in 4% paraformaldehyde prepared in PBS containing 0.03% Triton X‐100, followed by rinsing in PBS supplemented with 0.1% Tween‐20. Permeabilization was then carried out by incubating the sections with Proteinase K (20 µg/mL) for 15 min at ambient temperature. After a second round of fixation and washing, samples were equilibrated in hybridization buffer for 30 min at 37°C, and subsequently hybridized overnight at the same temperature with the appropriate probe mixture (25 pmol per probe). In dual‐labeling assays, two gene‐specific probes (25 pmol each) were combined prior to hybridization.

Excess probes (Data S14) were removed by four sequential 15‐min washes in pre‐warmed wash buffer, followed by two 10‐min rinses in 5× SSC containing 0.1% Tween‐20 at room temperature. Sections were then equilibrated in amplification buffer for 10 min. Fluorophore‐tagged hairpins were assigned as follows: *Rh50* (Alexa Fluor 488), *Amt* (Alexa Fluor 647). For dual detection, hairpins carrying two spectrally distinct fluorophores were pooled and applied together. Hairpin stocks (18 pmol each) were snap‐cooled prior to use and incubated on the sections overnight at room temperature in the dark. Finally, slides were rinsed in 5×SSC with 0.1% Tween‐20 and coverslipped using a DAPI‐containing antifade mounting medium (P0131, Beyotime Biotechnology, China). Fluorescence images were captured with a ZEISS LSM 900 confocal laser scanning microscope, and image processing was performed in ZEISS ZEN 3.9 software.

### 4.9 Ambient RNA Correction by Soupx

Ambient RNA contamination was evaluated and corrected using SoupX [75], (V.1.6.2). For each sex, the raw and filtered 10× feature‐barcode matrices were used to estimate the ambient RNA profile and contamination fraction. SoupX correction was performed separately for the female and male libraries at the whole‐library count‐matrix level. The corrected count matrices were then matched back to neuronal atlas metadata to recover ORN subcluster annotations. *BdorAmt/BdorOrco* transcript*‐co‐*detected nuclei were re‐identified after correction, and their enrichment in ORN16 relative to other ORN populations was tested using Fisher's exact test (Data S11).

### 4.10 Establish the *BdorAmt* Mutant Lines

*Amt* knockout mutants were generated following previously reported methods [76], including sgRNA synthesis, embryo microinjection, and mutant screening. Genomic DNA was extracted from the mid‐legs of adult flies, and candidate target regions were amplified using specific primers. The PCR products were cloned into a blunt‐end vector (pEASYBlunt Cloning Kit, TransGen Biotech, Beijing, China). Subsequently, 20 individual bacterial colonies were screened for each amplicon to identify conserved regions within the genes for the purpose of target design. Two sgRNAs were designed across the second exon within the CDS of *BdorAmt*. All sgRNAs were designed using CRISPOR [77] (https://crispor.gi.ucsc.edu/crispor.py) and consisted of 20‐bp target sites adjacent to an NGG (or CCN on the opposite strand) PAM. To ensure specificity, candidate sgRNAs (20‐nt protospacers with adjacent PAM) were aligned to the *B. dorsalis* telomere‐to‐telomere (t2t) reference genome [42] using BLASTn (task = blastn‐short). sgRNAs were synthesized using a commercial kit (GeneArt gRNA Kit, Thermo Fisher Scientific).

After sgRNA design and synthesis, we proceeded with embryo collection and microinjection in *B. dorsalis*. Adult insects aged 9–12 days were kept in transparent cages and provided with sufficient food and water. The insects were allowed to mate for 3 days, after which an embryo collection apparatus containing orange juice was introduced. Embryos collected within a 30‐min window were used for injections. A mixture of sgRNAs (sgRNA1+2) was injected at a working concentration of 300 ng/µL for sgRNA and 150 ng/µL for Cas9 protein. Embryo injections were performed using the FemtoJet and InjectMan 4 systems (Eppendorf, Hamburg, Germany). Injected embryos were incubated at 26.5°C and 60% relative humidity. Hatched larvae were transferred with a soft brush to an artificial diet and reared under standard laboratory conditions.

Genomic DNA was extracted from the mid‐legs of mutant adult flies for genotyping via the specific primers and PCR conditions described above. PCR‐amplified fragments were sequenced via the Sanger platform, and individuals with overlapping peaks at the target site were identified as carriers of edited genes. The PCR products were subsequently cloned and inserted into blunt‐end vectors for precise genotype determination. Adult G0 flies were backcrossed with wild‐type flies, and G1 offspring were genotyped to isolate heterozygous mutants. The desired G1 heterozygotes were selected and crossed with wild‐type flies to expand the population and generate G2 heterozygotes, which were then intercrossed to yield G3 homozygotes. G3 homozygous lines were established and maintained for further experiments. Ultimately, two independent frameshift mutant lines, each predicted to cause premature termination of the *Amt‐*encoded protein, were retained for downstream analyses. We evaluated potential off‐target effects by designing sgRNAs with CRISPOR (https://crispor.gi.ucsc.edu/crispor.py) and further checking candidate target sites against the local *B. dorsalis* genome assembly using BLAST. Only sgRNAs with unique genomic target sites were selected. In addition, two independent *BdorAmt* mutant lines showed consistent behavioral phenotypes, reducing the likelihood that the observed effects were caused by line‐specific off‐target mutations.

### 4.11 The Assessment of Ammonia Releasing From Bird Dropping

The ammonia release from bird droppings is assessed using Nessler's reagent (WEIYEL Inc, China) spectrophotometry [78] (Examination Methods for Public Places—Part 2: Chemical Pollutants, GBT18204.2‐2014). Briefly, 0.1 g of each sample group is placed in a vacuum filtration apparatus, with dilute sulfuric acid (0.005 mol/L) used as the ammonia absorption solution. The extraction is set at a flow rate of 0.4 L/min for 5 min. The ammonia‐containing dilute sulfuric acid reacts with Nessler's reagent, and the resulting color intensity is quantified at a wavelength of 425 nm using a spectrophotometer. The specific ammonia content is determined using standard ammonia working solutions as a quantitative standard, with three biological replicates conducted.

### 4.12 Olfactory Trap Assay

The behavioral responses of 12‐day‐old unmated male and female adults to ammonia were evaluated using a two‐choice trap assay. Ammonia odor was delivered as an ammonium hydroxide solution at concentrations of 0.25%, 0.5%, and 1% (v/v), with a loading volume of 500 µL per trap. The trap consisted of a conical flask (mouth diameter 3 cm, bottom diameter 5 cm, height 7.5 cm) fitted with a 1000‐µL pipette tip as the entrance, which allowed insect entry but prevented escape. The trap was placed inside a cage measuring 18 cm × 24 cm × 14 cm (length × width × height). Males and females were reared separately in small cages (30–40 adults per cage) prior to testing, and food was replaced every 2 days with water provided. Before the assay, adults were allowed to acclimate to the experimental cage for 20 min. The experiment was conducted from 17:00 to 20:00 under controlled conditions (∼26°C, ∼60% relative humidity, 60 lux). Each treatment consisted of a trap baited with the ammonia solution and a control trap baited with an equal volume of solvent. After the assay, the number of adults captured in each trap was recorded. Five replicates were performed, each using 30 adults. The attraction index (AI) was calculated as the number of adults trapped in the treatment trap minus the number trapped in the control trap, divided by the total number of adults used in the assay.

### 4.13 Survival and Reproduction Assays

Survival and reproduction assays were used to evaluate the effects that bird droppings as an additional nutrient.

For the survival assay, we used the life curve method to compare the survival differences between males and females when fed with bird droppings as a nutrient source. Forty unmated adults were maintained in a transparent cage (18 cm × 12.5 cm × 14 cm) with either additional nutrients or sugar as a control. The rearing conditions were set at 26±1°C, with relative humidity at 60±5% and a light‐dark cycle of 14L:10D. Observations were recorded for 50 days with 3 biological replicates (each contained 40 individuals that were tested together as a single cohort).

For the reproduction assay, we first evaluated the differences in mating success between males and females when fed with bird droppings as a nutrient source. Male and female adults were reared under the aforementioned conditions with either additional nutrients (bird droppings) or sugar as a control until they reached 12 days of age. The treated adults were then paired with untreated partners (1:1) in mating cages (18 cm × 12.5 cm × 14 cm). Mating events were recorded every 30 min during the observation period from 16:30 to 22:00. Observations were recorded for 5 biological replicates, each containing 20 individuals. Additionally, ovaries were dissected from females supplemented with bird droppings and those from the control group. Ovarian size (length and width) was assessed using n = 10 biological replicates per group, with each replicate derived from a single ovary. Egg load, defined as the number of eggs per ovary, was quantified using a separate set of n = 10 biological replicates, with each replicate also representing a single ovary. Oviposition performance was evaluated using n = 5 biological replicates, with each replicate consisting of the total number of eggs laid by 10 females.

### 4.14 Statistical Analysis

Cell clustering used throughout the study was performed with the Louvain algorithm implemented in Seurat after Harmony integration. To assess the robustness of the clustering results, we additionally performed clustering using the Leiden algorithm with the same Harmony embeddings and clustering resolution. Information on the versions of the R packages used in analysis of snRNA‐seq is provided in Data S13. For numerical experimental data, analyses were performed on the original values without data transformation, and no data points were excluded as outliers. The normality of the original data was initially assessed using the Shapiro‐Wilk test. Unless otherwise stated, experimental data are presented as mean ± SEM. Survival data are presented as Kaplan–Meier survival curves with 95% confidence intervals. The sample size (n) for each experiment is provided in the experimental design and corresponding figure legends. Normally distributed data were analyzed using the unpaired *t*‐test (for 2 groups) or one‐way ANOVA (for multiple groups, followed by Tukey's multiple comparisons test), whereas non‐normally distributed data were analyzed using the Mann‐Whitney *U* test (for 2 groups) or Kruskal‐Wallis test (for multiple groups, followed by Dunn's multiple comparisons test). For bounded index data, such as the attraction index, Wilcoxon rank‐sum tests were used for two‐group comparisons. For multiple pairwise comparisons, pairwise Wilcoxon rank‐sum tests were performed, with *p* values adjusted using the Benjamini–Hochberg method. All statistical tests were two‐sided unless otherwise stated. A *p* value of less than 0.05 was considered to indicate statistical significance. These statistical analyses were performed using GraphPad Prism (Version 10.4.0) (non‐index data) or R (version 4.5.2) (index data). For survival analysis, the data were analyzed using the Kaplan–Meier method with the ‘survival’ and ‘survminer’ packages in R (version 4.4.3).

## Author Contributions

W.L., J.X., and G.R.W. conceptualized and designed the experiments. W.L., S.Z., J.Z., Z.T., and L.Y. performed most of experiments. Y.B.X. performed FASC sorting. S.Z. performed the bioinformatics analyses. W.L. and J.Z. performed insect KO experiments. W.L., J.X., and G.R.W. analyzed the data. W.L. and J.X. wrote the first draft of the paper. G.R.W., J.X and W.L. edited the paper. All authors discussed the results and commented on the paper.

## Funding

This work was funded by the Financial Support for Outstanding Scientific and Technological Innovation Talents Training Fund in Shenzhen, the Shenzhen Science and Technology Program (Grant No. KCXFZ20240903093859009), the National Key Research and Development Program of China (Grant No. 2022YFD1700200), the IAEA Coordinated Research Project D41031, D44006 and the CAS Pioneer Hundred Talents Program.

## Conflicts of Interest

The authors declare no conflicts of interests.

## Declaration of Generative Ai

During the preparation of this manuscript, the authors used OpenAI's ChatGPT and Google's Gemini to assist in generating custom Python/R scripts for the analysis of prediction results and to improve the readability and language of the text. After using these tools, the authors reviewed and edited the content as needed and take full responsibility for the publication.

## Data and Materials Availability

The data that support the findings of this study are openly available and are included in Dataset of telomere‐to‐telomere assembly at https://ngdc.cncb.ac.cn/bioproject/browse/PRJCA031569.

## Supporting information




**Supporting File 1**: advs77168‐sup‐0001‐SuppMat.pdf.

**Supporting File 2**: advs77168‐sup‐0002‐SuppMat.zip.

**Supporting File 3**: advs77168‐sup‐0003‐SuppMat.docx.
