# Supplementary material for: Asymmetric Evolution of Antennal Cell Types Underlies a Derived Ammonia‐Sensing Logic for Ecological Adaptation in Bactrocera dorsalis
Source: Adv Sci (Weinh). 2026 Aug 17;13(62):e77168. doi: 10.1002/advs.77168 (PMC13479590; doi:10.1002/advs.77168)
Supplement: Supplementary file 1 [file ADVS-13-e77168-s001.pdf]

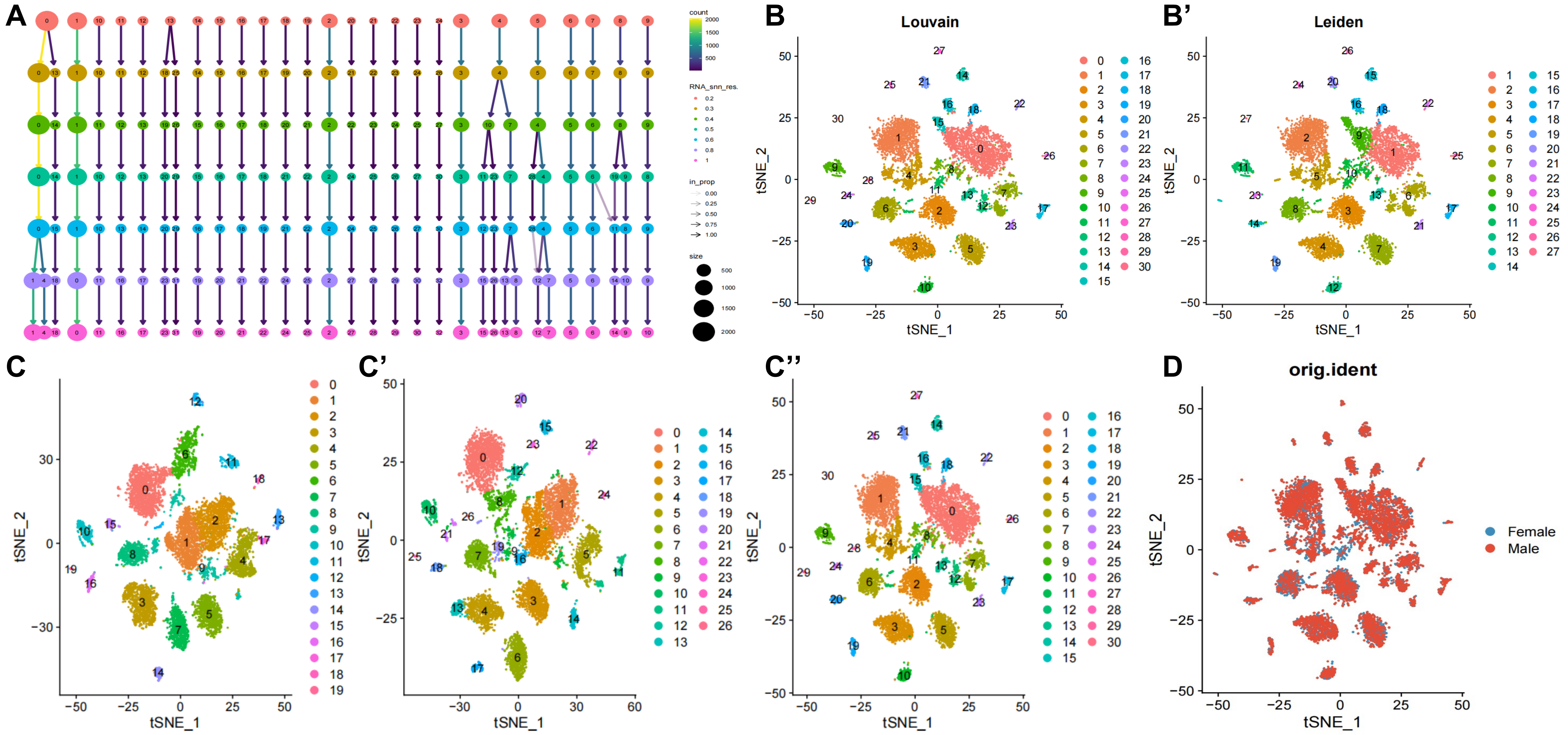
A
count
2000
1500
1000
500
RNA_snn_res.
0.2
0.3
0.4
0.5
0.6
0.8
1
in_prop
0.00
0.25
0.50
0.75
1.00
size
500
1000
1500
2000
B
Louvain
B'
Leiden
C
C'
C''
D
orig.ident
Female
Male
tSNE_1
tSNE_2

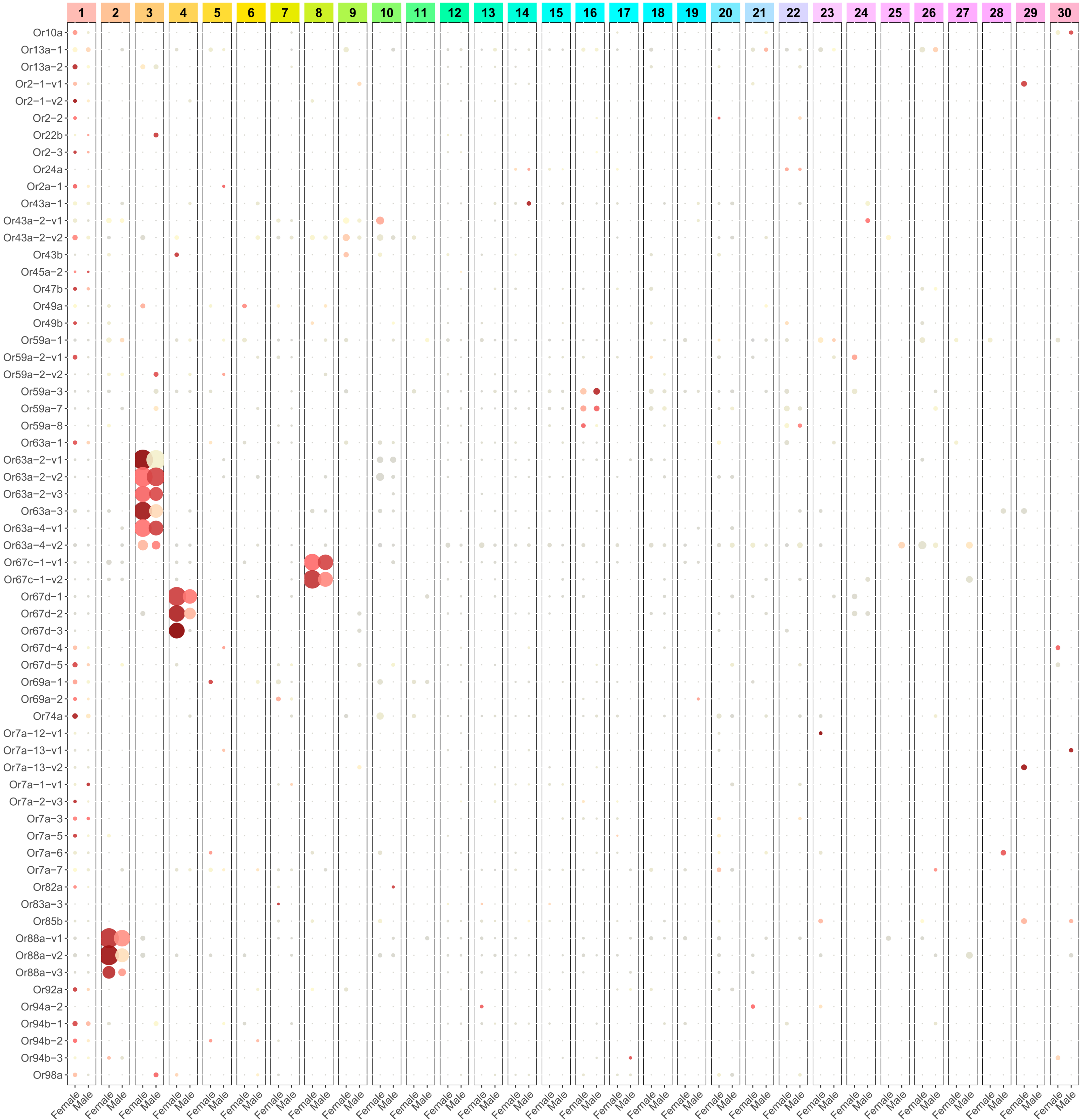

number	Cell cluster
1	adult olfactory co-receptor neuron
2	adult olfactory receptor neuron Or88a
3	adult olfactory receptor neuron Or63a
4	adult olfactory receptor neuron Or67d
5	adult olfactory receptor neuron Ir75d
6	adult olfactory receptor neuron Ir76a, Ir76b
7	adult olfactory receptor neuron Ir84a, Ir8a
8	adult olfactory receptor neuron Or67c
9	adult olfactory receptor neuron Gr21a/63a
10	johnston organ neuron
11	lamina monopolar neuron L4
12	epithelial cell-1
13	epithelial cell-2
14	support cell-1
15	support cell-2
16	support cell-3
17	support cell-4
18	support cell-5
19	support cell-6
20	support cell-7
21	support cell (glia)-1
22	support cell (glia)-2
23	support cell (glia)-3
24	support cell (glia)-4
25	muscle cell
26	hemocyte
27	unannotated-1
28	unannotated-2
29	unannotated-3
30	unannotated-4

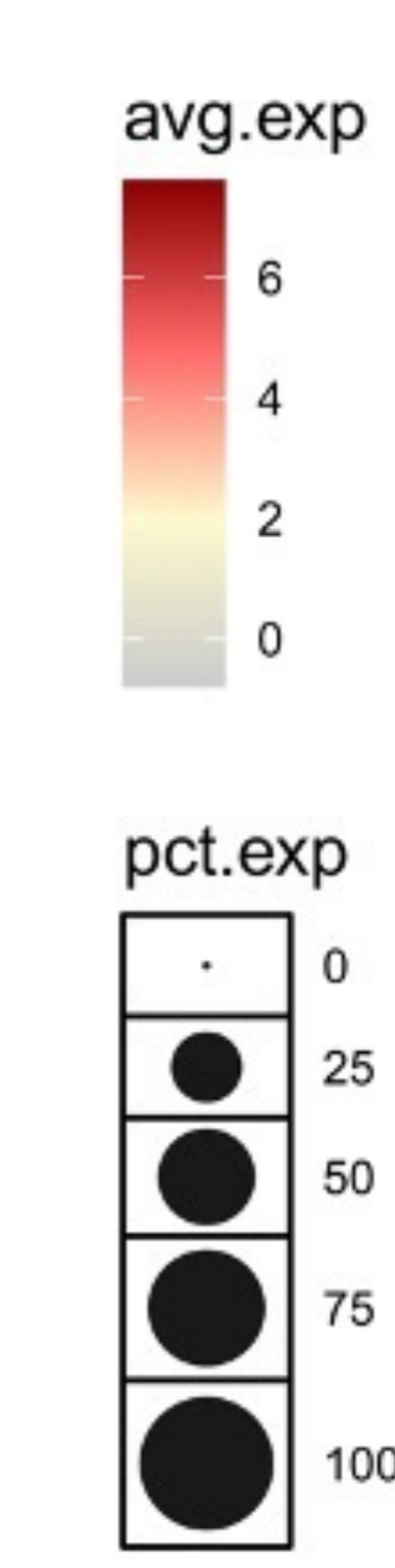

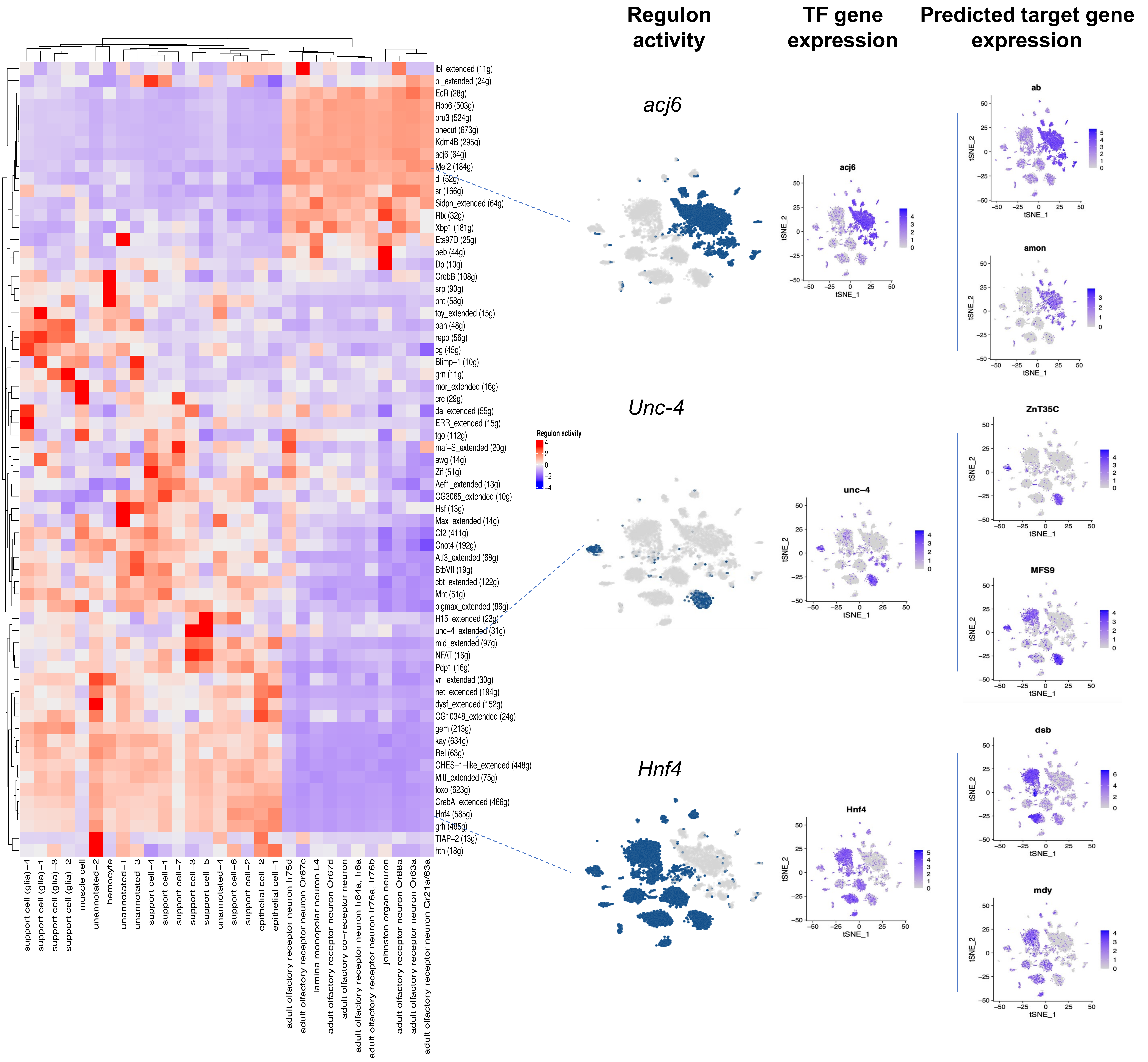
Regulon activity
TF gene expression
Predicted target gene expression
lbl_extended (11g)
bi_extended (24g)
EcR (28g)
Rbp6 (503g)
bru3 (524g)
onecut (673g)
Kdm4B (295g)
acj6 (64g)
Mef2 (184g)
dl (52g)
sr (166g)
Sidpn_extended (64g)
Rfx (32g)
Xbp1 (181g)
Ets97D (25g)
peb (44g)
Dp (10g)
CrebB (108g)
srp (90g)
pnt (58g)
toy_extended (15g)
pan (48g)
repo (56g)
cg (45g)
Blimp−1 (10g)
grn (11g)
mor_extended (16g)
crc (29g)
da_extended (55g)
ERR_extended (15g)
tgo (112g)
maf−S_extended (20g)
ewg (14g)
Zif (51g)
Aef1_extended (13g)
CG3065_extended (10g)
Hsf (13g)
Max_extended (14g)
Cf2 (411g)
Cnot4 (192g)
Atf3_extended (68g)
BtbVII (19g)
cbt_extended (122g)
Mnt (51g)
bigmax_extended (86g)
H15_extended (23g)
unc−4_extended (31g)
mid_extended (97g)
NFAT (16g)
Pdp1 (16g)
vri_extended (30g)
net_extended (194g)
dysf_extended (152g)
CG10348_extended (24g)
gem (213g)
kay (634g)
Rel (63g)
CHES−1−like_extended (448g)
Mitf_extended (75g)
foxo (623g)
CrebA_extended (466g)
Hnf4 (585g)
grh (485g)
TfAP−2 (13g)
hth (18g)
support cell (glia)−4
support cell (glia)−1
support cell (glia)−3
support cell (glia)−2
muscle cell
unannotated−2
hemocyte
unannotated−1
unannotated−3
support cell−4
support cell−1
support cell−7
support cell−3
support cell−5
unannotated−4
support cell−6
support cell−2
epithelial cell−2
epithelial cell−1
adult olfactory receptor neuron Ir75d
adult olfactory receptor neuron Or67c
lamina monopolar neuron L4
adult olfactory receptor neuron Or67d
adult olfactory co−receptor neuron
adult olfactory receptor neuron Ir84a, Ir8a
adult olfactory receptor neuron Ir76a, Ir76b
johnston organ neuron
adult olfactory receptor neuron Or88a
adult olfactory receptor neuron Or63a
adult olfactory receptor neuron Gr21a/63a
Regulon activity
4
2
0
−2
−4
acj6
Unc-4
Hnf4
acj6
unc−4
Hnf4
ab
amon
ZnT35C
MFS9
dsb
mdy
tSNE_1
tSNE_2

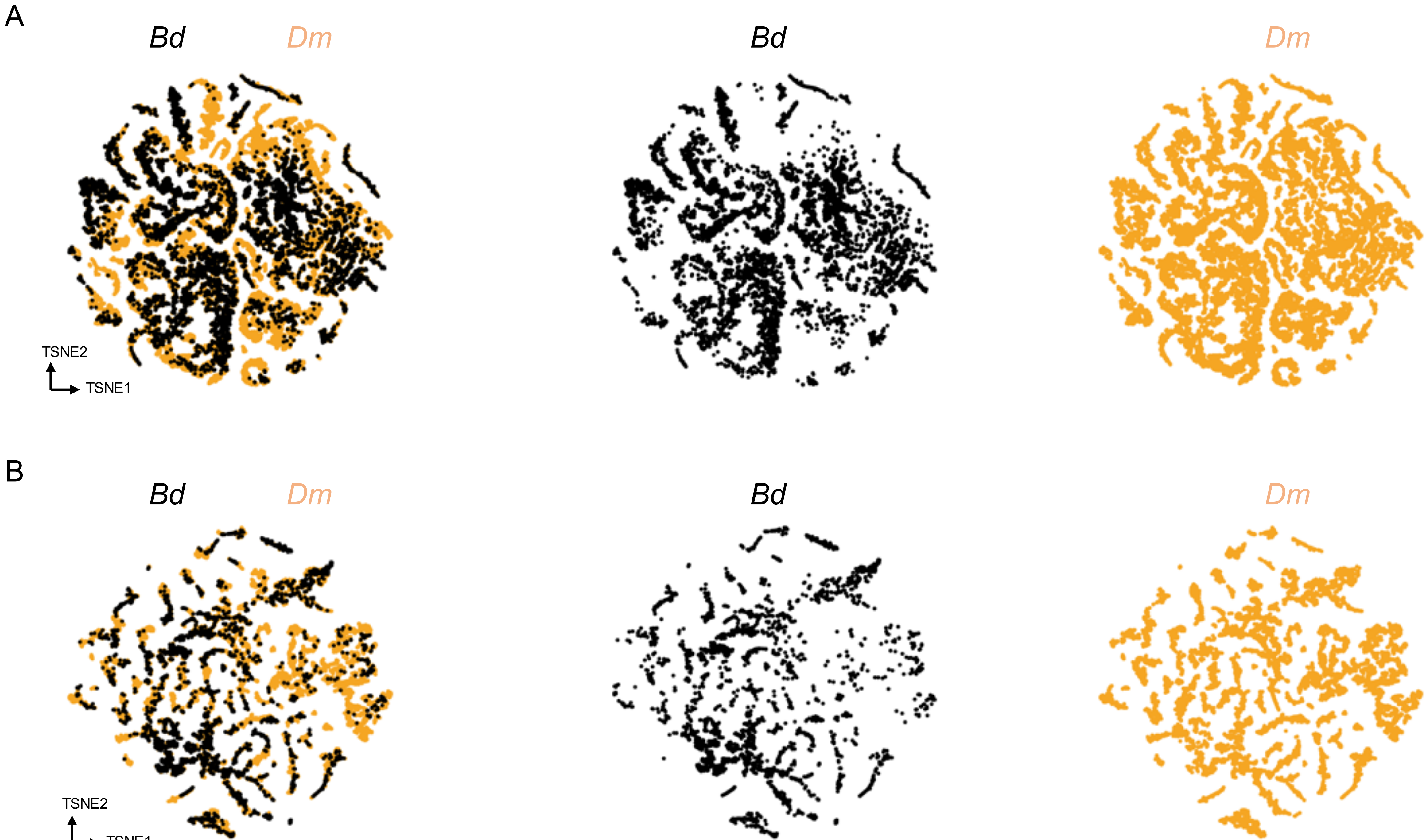
A
Bd
Dm
Bd
Dm
TSNE2
TSNE1
B
Bd
Dm
Bd
Dm
TSNE2
TSNE1

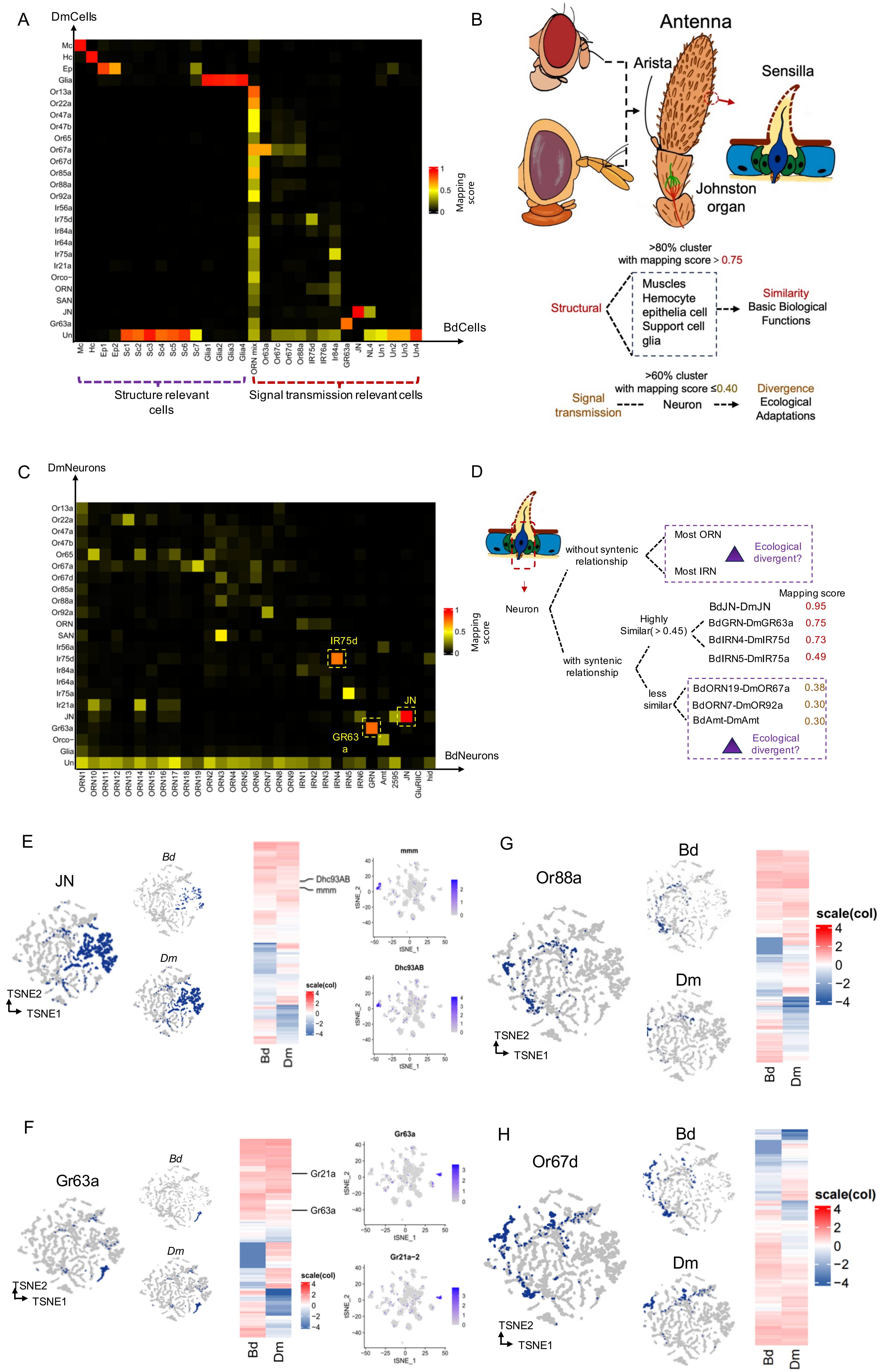

A
DmCells
Mc
Hc
Ep
Glia
Or13a
Or22a
Or47a
Or47b
Or65
Or67a
Or67d
Or85a
Or88a
Or92a
Ir56a
Ir75d
Ir84a
Ir64a
Ir75a
Ir21a
Orco-
ORN
SAN
JN
Gr63a
Un
Mc
Hc
Ep1
Ep2
Sc1
Sc2
Sc3
Sc4
Sc5
Sc6
Sc7
Glia1
Glia2
Glia3
Glia4
ORN mix
Or63a
Or67c
Or67d
Or88a
IR75d
IR76a
Ir84a
GR63a
JN
NL4
Un1
Un2
Un3
Un4
BdCells
Mapping score
1
0.5
0
Structure relevant cells
Signal transmission relevant cells
B
Antenna
Arista
Sensilla
Johnston organ
>80% cluster with mapping score > 0.75
Structural
Muscles
Hemocyte
epithelia cell
Support cell
glia
Similarity
Basic Biological Functions
>60% cluster with mapping score ≤0.40
Signal transmission
Neuron
Divergence
Ecological Adaptations
C
DmNeurons
Or13a
Or22a
Or47a
Or47b
Or65
Or67a
Or67d
Or85a
Or88a
Or92a
ORN
SAN
Ir56a
Ir75d
Ir84a
Ir64a
Ir75a
Ir21a
JN
Gr63a
Orco-
Glia
Un
ORN1
ORN10
ORN11
ORN12
ORN13
ORN14
ORN15
ORN16
ORN17
ORN18
ORN19
ORN2
ORN3
ORN4
ORN5
ORN6
ORN7
ORN8
ORN9
IRN1
IRN2
IRN3
IRN4
IRN5
IRN6
GRN
Amt
2595
JN
GluRIIC
hid
BdNeurons
IR75d
JN
GR63a
Mapping score
1
0.5
0
D
without syntenic relationship
Most ORN
Most IRN
Ecological divergent?
Neuron
Mapping score
Highly Similar(> 0.45)
BdJN-DmJN 0.95
BdGRN-DmGR63a 0.75
BdIRN4-DmIR75d 0.73
BdIRN5-DmIR75a 0.49
with syntenic relationship
less similar
BdORN19-DmOR67a 0.38
BdORN7-DmOR92a 0.30
BdAmt-DmAmt 0.30
Ecological divergent?
E
JN
Bd
Dm
TSNE2
TSNE1
Dhc93AB
mmm
scale(col)
Bd
Dm
mmm
Dhc93AB
tSNE_1
tSNE_2
F
Gr63a
Bd
Dm
TSNE2
TSNE1
Gr21a
Gr63a
scale(col)
Bd
Dm
Gr63a
Gr21a-2
tSNE_1
tSNE_2
G
Or88a
Bd
Dm
TSNE2
TSNE1
scale(col)
4
2
0
−2
−4
Bd
Dm
H
Or67d
Bd
Dm
TSNE2
TSNE1
scale(col)
4
2
0
−2
−4
Bd
Dm

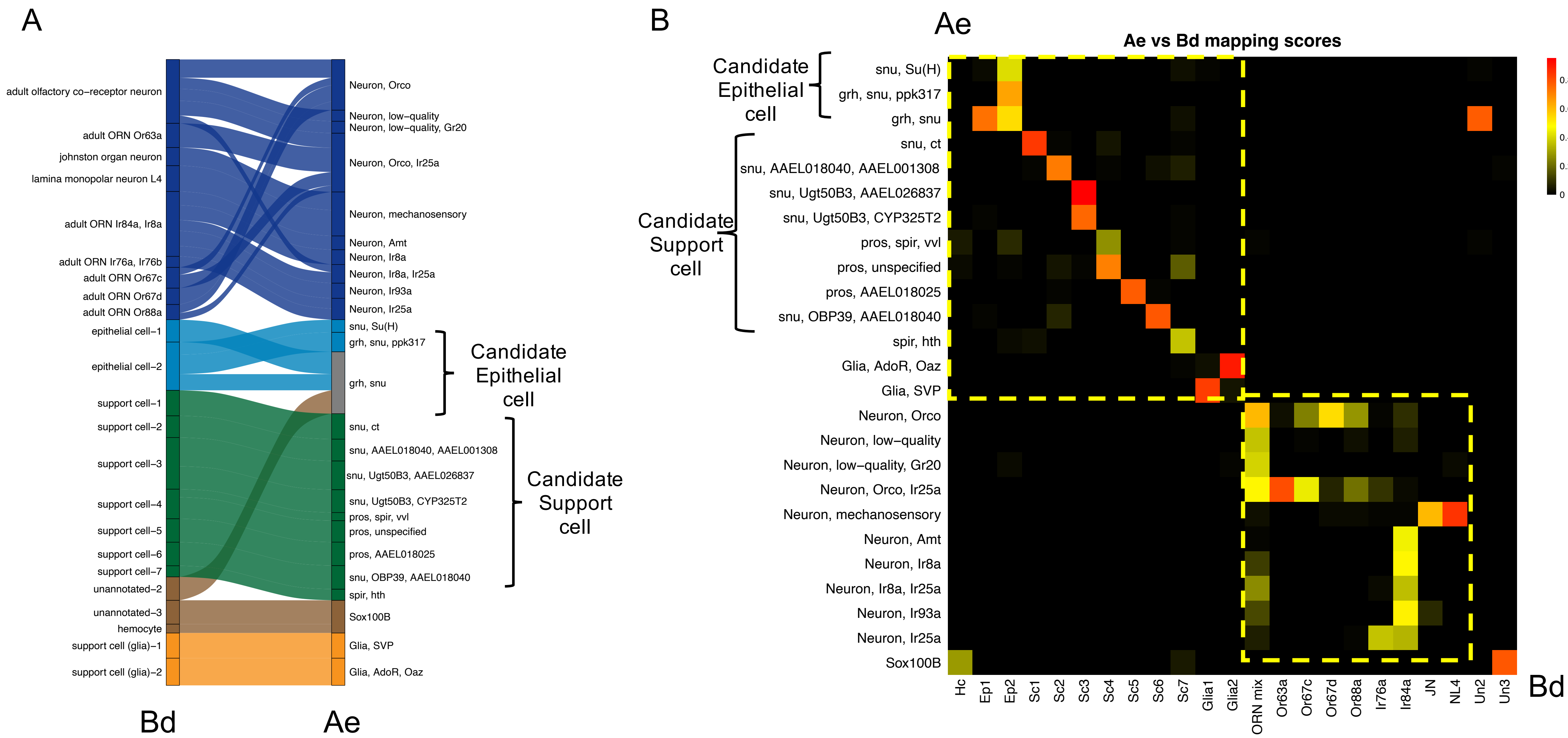
A
B
Bd
Ae
adult olfactory co−receptor neuron
adult ORN Or63a
johnston organ neuron
lamina monopolar neuron L4
adult ORN Ir84a, Ir8a
adult ORN Ir76a, Ir76b
adult ORN Or67c
adult ORN Or67d
adult ORN Or88a
epithelial cell−1
epithelial cell−2
support cell−1
support cell−2
support cell−3
support cell−4
support cell−5
support cell−6
support cell−7
unannotated−2
unannotated−3
hemocyte
support cell (glia)−1
support cell (glia)−2
Neuron, Orco
Neuron, low−quality
Neuron, low−quality, Gr20
Neuron, Orco, Ir25a
Neuron, mechanosensory
Neuron, Amt
Neuron, Ir8a
Neuron, Ir8a, Ir25a
Neuron, Ir93a
Neuron, Ir25a
snu, Su(H)
grh, snu, ppk317
grh, snu
snu, ct
snu, AAEL018040, AAEL001308
snu, Ugt50B3, AAEL026837
snu, Ugt50B3, CYP325T2
pros, spir, vvl
pros, unspecified
pros, AAEL018025
snu, OBP39, AAEL018040
spir, hth
Sox100B
Glia, SVP
Glia, AdoR, Oaz
Candidate Epithelial cell
Candidate Support cell
Ae vs Bd mapping scores
0.8
0.6
0.4
0.2
0
Hc
Ep1
Ep2
Sc1
Sc2
Sc3
Sc4
Sc5
Sc6
Sc7
Glia1
Glia2
ORN mix
Or63a
Or67c
Or67d
Or88a
Ir76a
Ir84a
JN
NL4
Un2
Un3

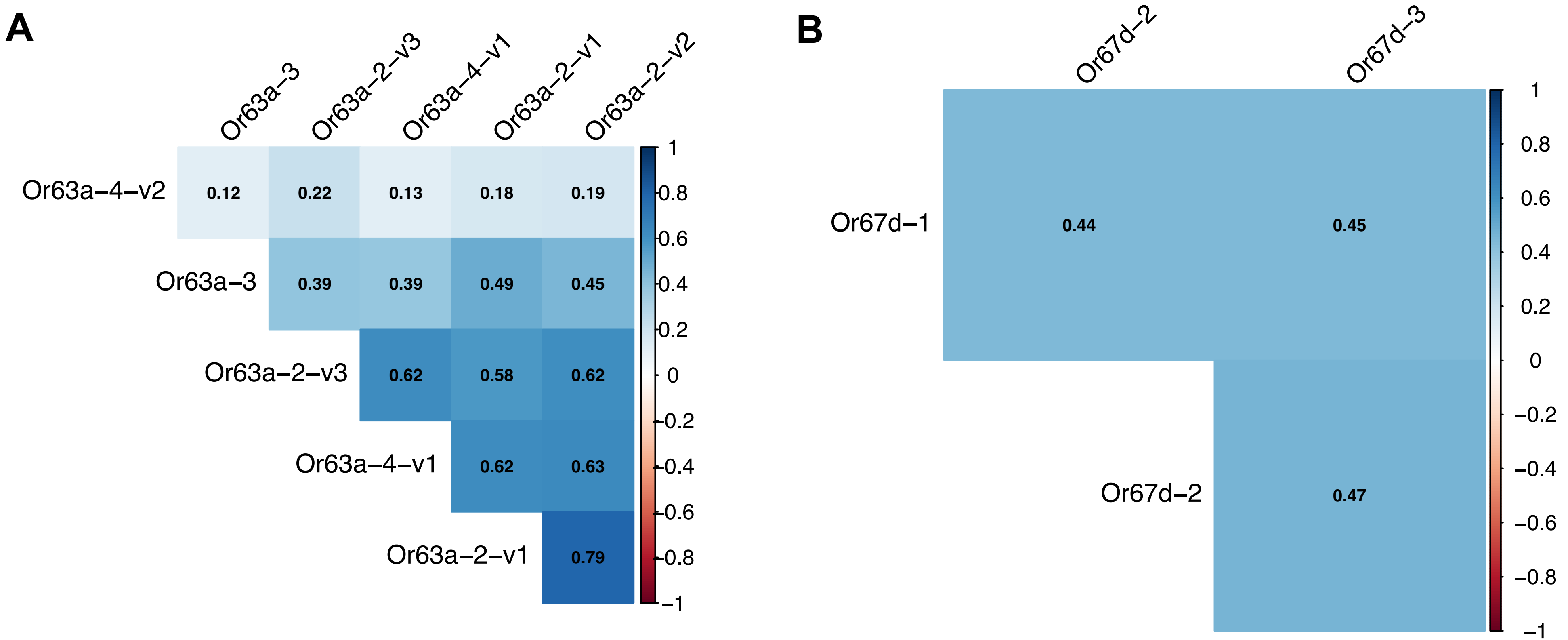
A
Or63a−3
Or63a−2−v3
Or63a−4−v1
Or63a−2−v1
Or63a−2−v2
Or63a−4−v2
0.12
0.22
0.13
0.18
0.19
Or63a−3
0.39
0.39
0.49
0.45
Or63a−2−v3
0.62
0.58
0.62
Or63a−4−v1
0.62
0.63
Or63a−2−v1
0.79
1
0.8
0.6
0.4
0.2
0
−0.2
−0.4
−0.6
−0.8
−1
B
Or67d−2
Or67d−3
Or67d−1
0.44
0.45
Or67d−2
0.47
1
0.8
0.6
0.4
0.2
0
−0.2
−0.4
−0.6
−0.8
−1

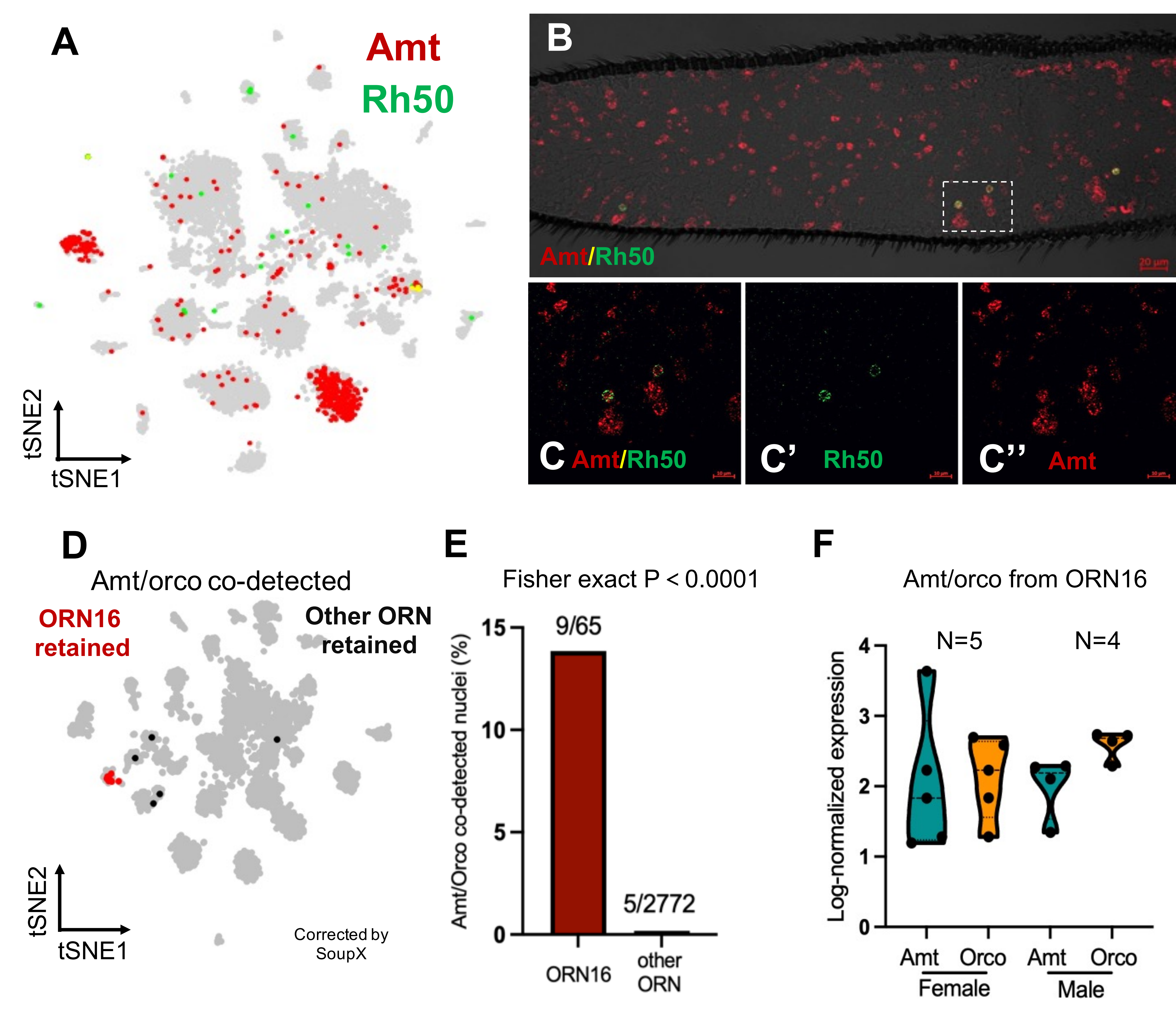
A
Amt
Rh50
tSNE2
tSNE1
B
Amt/Rh50
20 µm
C Amt/Rh50
C' Rh50
C'' Amt
D
Amt/orco co-detected
ORN16 retained
Other ORN retained
tSNE2
tSNE1
Corrected by SoupX
E
Fisher exact P < 0.0001
Amt/Orco co-detected nuclei (%)
15
10
5
0
9/65
5/2772
ORN16
other ORN
F
Amt/orco from ORN16
Log-normalized expression
4
3
2
1
0
N=5
N=4
Amt
Orco
Amt
Orco
Female
Male

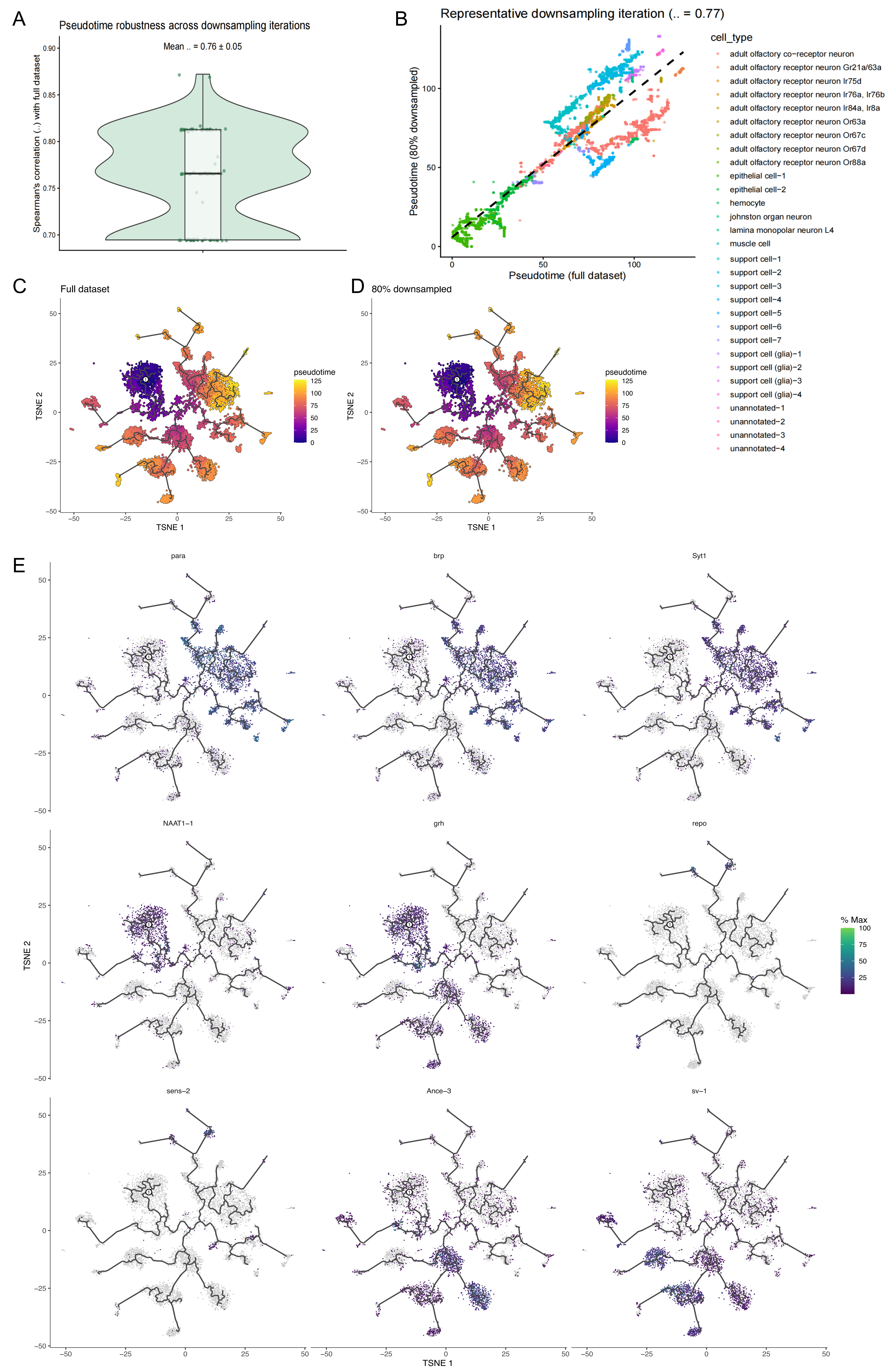
A
Pseudotime robustness across downsampling iterations
Mean .. = 0.76 ± 0.05
Spearman's correlation (..) with full dataset
0.90
0.85
0.80
0.75
0.70
B
Representative downsampling iteration (.. = 0.77)
Pseudotime (80% downsampled)
Pseudotime (full dataset)
0
50
100
cell_type
adult olfactory co−receptor neuron
adult olfactory receptor neuron Gr21a/63a
adult olfactory receptor neuron Ir75d
adult olfactory receptor neuron Ir76a, Ir76b
adult olfactory receptor neuron Ir84a, Ir8a
adult olfactory receptor neuron Or63a
adult olfactory receptor neuron Or67c
adult olfactory receptor neuron Or67d
adult olfactory receptor neuron Or88a
epithelial cell−1
epithelial cell−2
hemocyte
johnston organ neuron
lamina monopolar neuron L4
muscle cell
support cell−1
support cell−2
support cell−3
support cell−4
support cell−5
support cell−6
support cell−7
support cell (glia)−1
support cell (glia)−2
support cell (glia)−3
support cell (glia)−4
unannotated−1
unannotated−2
unannotated−3
unannotated−4
C
Full dataset
D
80% downsampled
pseudotime
125
100
75
50
25
0
TSNE 1
TSNE 2
E
para
brp
Syt1
NAAT1−1
grh
repo
sens−2
Ance−3
sv−1
% Max
100
75
50
25
TSNE 1
TSNE 2

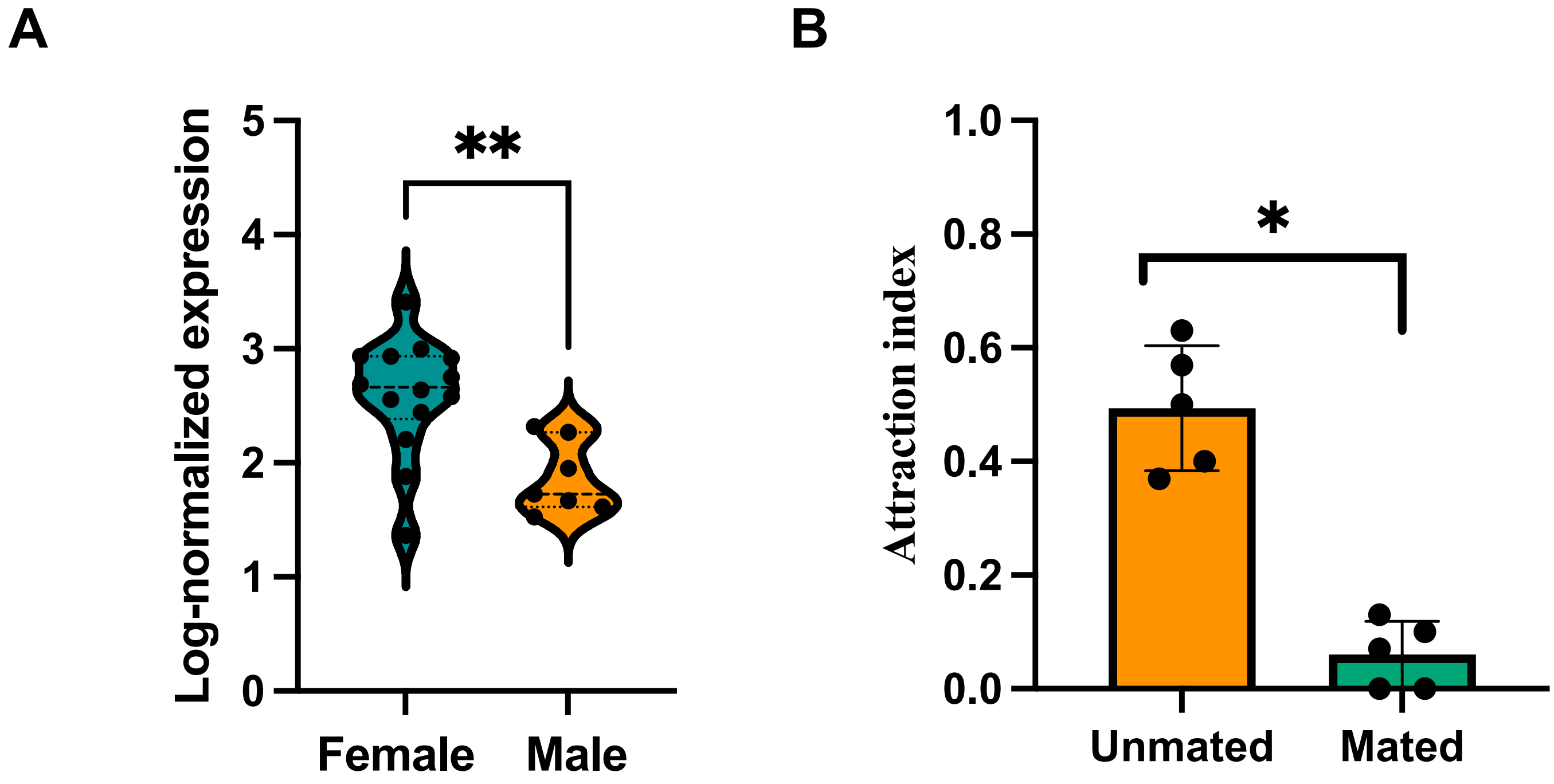
A
Log-normalized expression
5
4
3
2
1
0
**
Female
Male
B
Attraction index
1.0
0.8
0.6
0.4
0.2
0.0
*
Unmated
Mated
