# Supplementary material for: Asymmetric Evolution of Antennal Cell Types Underlies a Derived Ammonia‐Sensing Logic for Ecological Adaptation in Bactrocera dorsalis
Source: Adv Sci (Weinh). 2026 Aug 17:e77168. Online ahead of print. doi: 10.1002/advs.77168 (PMC13479590; doi:10.1002/advs.77168)
Supplement: Supplementary file 2 [file ADVS-9999-e77168-s002.docx]

**Supplementary Figure**

**Figure S1-related to Figure 1. Assessment of clustering robustness and batch integration**

(A) Clustering stability across different clustering resolutions. Clustering was performed using the Harmony-corrected principal components with clustering resolutions ranging from 0.2 to 1.0. The cluster tree illustrates the relationships between clusters identified at successive resolutions. Major cell populations remained stable across a broad range of clustering resolutions, while only small subpopulations underwent further subdivision at higher resolutions, indicating that the clustering results were robust to the choice of resolution parameter. (B-B’) Comparison of clustering results generated by the Louvain and Leiden algorithms. t-SNE map projections of the same Harmony-integrated dataset clustered using the Louvain (left) and Leiden (right) community detection algorithms with identical clustering parameters. The two algorithms produced highly consistent clustering results (Adjusted Rand Index, ARI = 0.88), demonstrating the robustness of the identified cell populations. (C-C’’) Evaluation of clustering robustness using different numbers of Harmony dimensions. Clustering and t-SNE map visualization were performed using 20, 30, and 40 Harmony dimensions. Comparable clustering patterns and cell population structures were observed across all parameter settings, indicating that the identified cell populations were robust to the choice of dimensionality used for downstream analysis. (D) Assessment of batch integration following Harmony correction. t-SNE map projection of cells colored by sample identity (female and male antennae) after Harmony integration. Cells from the two samples were well mixed across all major cell populations, indicating effective correction of batch effects while preserving biological variation.

**Figure S2-related to Figure 1.** Distribution of olfactory receptor gene expression in female and male antennae. Expression levels and percentage of cells expressing the *Or* genes in each cluster are shown as a dot plot.

**Figure S3-related to Figure 1.** Cell-type–specific gene regulatory landscape of *B. dorsalis* adult antenna. The heatmap shows the gene-expression level in each cluster. Low regulon activity is shown with blue color and high regulon activity is shown in red. t-SNE map depiction of regulon activity (“on-blue,” “off-gray”) and TF gene expression (blue scale) of *acj6* (neuron cells), *Unc-4* (support cell), and Hnf4. Examples of target gene expression of the *acj6* regulon (*ab* and *amon*), Unc-4 regulon (*ZnT35C* and *MFS9*), and *Hnf4* regulon (*dsb* and *mdy*) are shown in blue.

**Figure S4-related to Figure 3.** Cross-species analysis of *B. dorsalis* and *D. melanogaster* adult whole antenna and antenna neuronal cells using SAMap. (A) t-SNE map projection of the combined *B. dorsalis* and *D. melanogaster* adult whole antenna manifolds. (B) t-SNE map projection of the combined *B. dorsalis* and *D. melanogaster* adult antenna sub-neuronal cells manifolds.

**Figure S5-related to Figure 4.** Comparison of *B. dorsalis* and *D. melanogaster* antenna at single-cell resolution. (A) Mapping score of *B. dorsalis* (bottom) and *D. melanogaster* (left) antenna cell clusters. Cross-species mapping scores range from 0 to 1 and are represented by a color gradient from black (low correlation) to red (high correlation). Bd, *B. dorsalis*; Dm, *D. melanogaster*. All mapping scores are provided in Data S8. (B) Schematic diagram of antennal structures in *D. melanogaster* and *B. dorsalis*. (C) Mapping score of neuronal subclusters from *B. dorsalis* (bottom) and *D. melanogaster* (left). Correlation coefficients range from 0 to 1 and are represented by a color gradient from black (low correlation) to red (high correlation). (D) Schematic diagram of antennal sensilla structures in *D. melanogaster* and *B. dorsalis*. (E-F) Comparative analysis of JN and GR63a neurons cell clusters between *B. dorsalis* and *D. melanogaste*r. The marker genes corresponding to neuronal subclusters are summarized in Supplementary Data S7. (G-H) Comparative analysis of *Or88a* and *Or67d* neurons cell clusters between *B. dorsalis* and *D. melanogaster*. The full names of the cell cluster annotations are listed in Data S6.

**Figure S6-related Figure 4.** Comparison of *B. dorsalis* (Bd) and *Aedes aegypti* (Ae) antenna at single-cell resolution. (A) Sankey plot that summarizing the cell type mapping between *B. dorsalis* and *A. aegypti.* Pairwise correlations of *B. dorsalis* (bottom) and *A. aegypti* (left) antenna cell clusters. (B) Cross-species mapping scores range from 0 to 1 and are represented by a color gradient from black (low correlation) to red (high correlation). Bd, *B. dorsalis*; Ae, *A. aegypti*. All mapping scores are provided in Data S8

**Figure S7-related to Figure 3. Correlation matrices of co-detected Or63a and Or67d receptor genes.** (A) Pairwise Pearson correlation matrix of six Or63a receptor transcripts co-detected in adult ORN19. (B) Pairwise Pearson correlation matrix of three Or67d receptor genes transcripts co-detected in adult ORN6. Correlations were calculated using Seurat LogNormalize-normalized expression values. Values indicate Pearson’s r, and color intensity represents correlation strength.

**Figure S8 -related to Figure 4. Spatial and computational support for *BdorAmt*-associated candidate neuronal populations in adult *B. dorsalis* antennae.** (A) t-SNE visualization showing the distribution of *BdorAmt*- and *BdorRh50*-transcript-detected nuclei in the antennal snRNA-seq atlas. Red indicates *BdorAmt*-transcript-detected nuclei, green indicates *BdorRh50*-transcript-detected nuclei, and yellow indicates *BdorAmt/BdorRh50* transcript-co-detected nuclei. (B) Representative HCR RNA FISH image showing *BdorAmt* and *BdorRh50* signals in an adult *B. dorsalis* (female adult) antenna. The boxed region is enlarged in C–C’’. (C–C’’) Enlarged views of the boxed region in B, showing merged *BdorAmt/BdorRh50* signals (C), *BdorRh50* signal- Alexa Fluor 488 (C’), and *BdorAmt* signal- Alexa Fluor 647 (C’’). (D) t-SNE visualization of *BdorAmt/BdorOrco* transcript-co-detected nuclei retained after SoupX-based ambient RNA correction. Red dots indicate retained *BdorAmt/BdorOrco* transcript-co-detected nuclei in ORN16, and black dots indicate retained nuclei in other ORN populations. (E) *BdorAmt/BdorOrco* transcript-co-detected nuclei were significantly enriched in ORN16 after *SoupX* correction. Data are shown as the percentage of *BdorAmt*/*BdorOrco* transcript-co-detected nuclei among all nuclei in each ORN group. After correction, 9 of 65 ORN16 nuclei retained *BdorAmt/BdorOrco* transcript co-detection, whereas only 5 of 2772 nuclei in other ORN populations did so. Fisher’s exact test, P <0.0001. (F) Log-normalized *BdorAmt* and *BdorOrco* expression in retained *BdorAmt/BdorOrco* transcript-co-detected ORN16 nuclei from female and male antennal libraries. Data are shown as violin plots with individual data points. dotted lines indicate quartiles and dashed lines indicate medians. n = 5 female nuclei and 4 male nuclei.

**Figure S9-related to Figure 4. Robustness of pseudotime trajectory to cell downsampling.** (A) Distribution of Spearman’s correlation coefficients (ρ) between pseudotime values computed on 80% randomly downsampled cells and the full dataset, across 100 independent iterations (mean ρ = 0.76 ± 0.05). (B) Scatter plot comparing pseudotime values from a representative downsampling iteration (ρ closest to the median of all iterations) against pseudotime values from the full dataset, with cells colored by annotated cell type; the dashed line indicates the linear regression fit. (C-D) Representative comparison of trajectory topology between the full dataset and one downsampled iteration, demonstrating consistent branch structure and pseudotime ordering despite cell subsampling. (E)Spatial expression patterns of marker genes overlaid on the pseudotime trajectory.

**Figure S10 -related to Figure 4 and 5. Sex- and mating-status-associated differences in *BdorAmt* expression and ammonia attraction in *B. dorsalis*.** (A) Log-normalized *BdorAmt* expression in female and male *BdorAmt/BdorRh50* transcript-co-detected candidate cells. Data are shown as violin plots with individual data points; dotted lines indicate quartiles and dashed lines indicate medians. Unpaired t tests were used for female–male comparisons; n=14 female nuclei and 7 male nuclei; ** P＜0.01. (B) Attraction of unmated and mated *B. dorsalis* females to ammonia (mean ± SEM). Wilcoxon rank-sum tests were used for unmated–mated female comparisons; n=5; * P＜0.05.
